# The fluorescence mechanism of carbon dots based on the separation and identification of small molecular fluorophores†

**DOI:** 10.1039/d2ra00431c

**Published:** 2022-04-14

**Authors:** Bingchen Han, Xin Hu, Xinfeng Zhang, Xianju Huang, Mingzhe An, Xiao Chen, Dan Zhao, Jun Li

**Affiliations:** School of Pharmaceutical Sciences, South-Central Minzu University Wuhan China lijun-pharm@hotmail.com; Key Laboratory of Wuliangye-flavor Liquor Solid-state Fermentation, Wuliangye Yibin Co. 3Ltd Yibin China; Hubei Yaosheng Traditional Chinese Medicine Technology Co. Ltd Zhaoyang China

## Abstract

Carbon dots (CDs) have attracted much attention in theoretical researches and their practical applications due to their excellent optical properties, and many researchers discovered that flurophores play a very important role in synthesis process of CDs and the luminescence of prepared CDs. In this study, two CDs were pyrolysis with citric acid, *N*-acetyl-l-cysteine and glutathione derivatives as carbon sources. Four intermediate small molecules were separated from the prepared CDs through ultrafiltration and chromatography, and their chemical structures were determined. The formation process of CDs was monitored through identified small molecule intermediates and HPLC. It is speculated that the two CDs have the same formation pathway, including TPA (5-oxo-2,3-dihydro-5*H*-[1,3]thiazolo[3,2-*a*]pyridine-3,7-dicarboxylic acid) synthesis, fluorophore polymerization, carbon chain extension, and carbonization. It was also discovered that these two CDs have the same fluorescence properties, thiazolopyridone structure, and nitrogen–sulfur co-doped functional groups are important reasons for the mixed excitation dependence of CDs. This study would provide valuable theoretical basis for the studies on preparation of excellent CDs, raw material selection, and CDs formation mechanism.

## Abstract 2

This paper reports the formation process and fluorescence mechanism of synthetic carbon dots using citric acid, *N*-acetyl-l-cysteine and glutathione derivatives as carbon sources.

## Graphical abstract

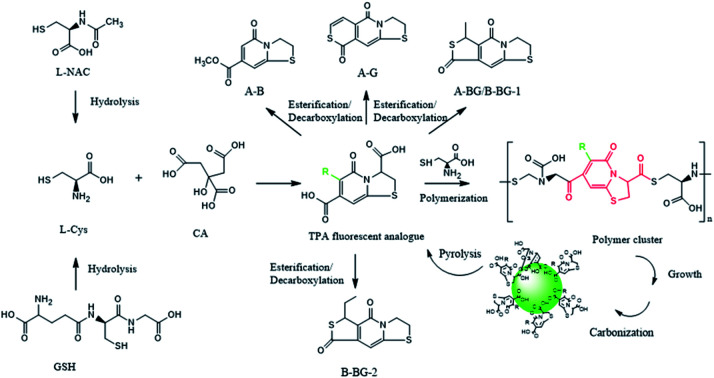

## 1. Introduction

Carbon dots(CDs) are nanoparticles with excellent luminescence properties and low toxicity, with many applications in biological imaging,^1–4^ drug delivery,^3^ photovoltaics, and catalysis,^5–7^ To broaden the application range of CDs, researchers have developed a variety of preparation methods for CDs for many years. Among them, the bottom-up preparation method have been widely used thanks to their efficient synthesis of multifunctional CDs,including pyrolysis,^8^ combustion,^9^ hydrothermal synthesis,^10,11^ and microwave synthesis.^12^

In 2008, Bourlinos^13^*et al.* reported the formation of fluorescent CDs from citric acid (CA) and alkyl amines through pyrolysis. Since then, CA has become a common and effective carbon source for the preparation of CDs.^14^ The pyrolysis preparation of CA has also been the preference of researchers because of its fluorescence characteristics and low cost. Though many reports on the synthetic routes, properties, and applications of CDs have been published, various reaction pathways between CA and nitrogen-containing compounds are possible in the process of hydrothermal or microwave synthesis, and their formation process still remains an open topic.

Currently, many formation mechanisms have been proposed.^14–18^ According to the references and previous work, the proposed formation process of CDs includes “polymerization”, “nucleation”, “carbonization” and “growth”. The fluorophores with diverse structures are firstly synthesized before the formation of CDs, and then the polymer carbon skeleton crosslinking fluorophores is formed by dehydration. In the process of carbonization, some fluorophores are consumed to further modify the carbon core. The small-molecule fluorophores play a very important role in these processes of CDs synthesis.

In addition, researchers also speculate that fluorophores are one of the factors that affect the luminescence properties of prepared CDs. For example, the surface state^18–22^ indicates that the synergistic hybridization between the carbon nucleus and the doping atom group formed by the polymerization of the fluorophore can produce surface defects, resulting in the emergence of new emission energy levels, and thus the appearance of different fluorescent colors. The molecular state^18^ reveals that the fluorescences, which can directly emit light, are the origins of CDs fluorescence when they are attached to the inside and outside of CDs. The carbon-nucleus state^18,23,24^ suggests that fluorescence originates from macromolecular polymers formed by the dehydration of initially formed fluorophores. Furthermore, the fluorophore also explains the excitation dependence property of CDs.^25,26^

Kasprzy^27^ identified the fluorophore TPA (5-oxo-2,3-dihydro-5*H*-[1,3]thiazolo[3,2-*a*]pyridine-3,7-dicarboxylic acid)by separation from the hydrolysate of biodegradable photoluminescent polyester (BPLP) and NMR, and discovered origin of the luminescence characteristics of BPLP. Song^28^ purified and isolated a bright blue fluorophore IPCA(imidazo[1,2-*a*]pyridine-7-carboxylic acid, 1,2,3,5-tetrahydro-5-oxo-) from synthetic CDs by chromatography in 2015 and identified its structure by NMR and proved that CDs contain complex components and multiple PL centers. The independent fluorophores on CDs would strongly affect PL characteristics of CDs. In addition, researchers also separated HPPT (4-hydroxy-1*H*-pyrrolo[3,4-*c*]pyridine-1,3,6(2*H*,5*H*)-trione)^29^ TPDCA(5-oxo-3,5-dihydro-2*H*-thiazolo [3,2-*a*]pyridine-3,7-dicarboxylic acid), and TPCA(5-oxo-3,5-dihydro-2*H*-thiazolo [3,2-*a*]pyridine-7-carboxylic acid)^30^ from CDs, and explained the luminescence mechanism of CDs. The results also confirmed that the elucidation of the chemical structure of fluorophores could help to understand the formation mechanism of CDs, as well as the composition, structure and photoluminescence origin of CDs.

In summary, since CDs are polymers with small molecular fluorophores as basic units, the structural identification of fluorophores that have not been polymerized or fallen off from CDs, would provide simple, quick and accurate exploration on the formation mechanism and fluorescence mechanism of prepared CDs. Therefore, the separation of fluorophores is of great importance.

In this paper, with CA, *N*-acetyl-l-cysteine (l-NAC), and glutathione (GSH) as carbon sources, two CDs (CDs CDs-NAC and CDs-GSH) have been prepared. The blue, blue-green, and green fluorophores were separated from prepared CDs by several separation methods, including ultrafiltration, column chromatography and HPLC. With the help of NMR and ESI-HRMS, the structures of the separated fluorophores were accurately characterized, and the impacts of difference raw materials upon the formation of fluorophores was studied. HPLC, UV-Vis, and fluorescence detections have been used to assist the analysis of the formation process of CDs, and discovered the same four stages of formation processes of these two types of CDs, that is, TPA synthesis, fluorophore polymerization, carbon chain extension, and carbonization. More importantly, TPA amide α-C generated during the reaction would turn into diverse fluorophores (TPA/TPA analogs) by connecting different groups, and these different fluorophores can be dehydrated and polymerized into different CDs. The discovery is of great significance when using CA and cysteine analogs as carbon sources to design CDs with richer fluorescence colours. Furthermore, the source of fluorescence emission sites of CDs has also been investigated and the existence of fluorophores has been found as the reason for CDs' excitation dependence property.

## 2. Experimental procedures

### 2.1 Chemicals

l-NAC (≥99.0%) and GSH (98.0%) were purchased from Shanghai Yuanye Bio-Technology Co., Ltd Methanol (≥99.5%), dichloromethane (≥99.5%), ethyl acetate (≥99.5%), and petroleum ether were purchased from Shanghai Titan Scientific Co. Ltd. Hexane (≥97.0%) and trifluoroacetic acid (TFA) were purchased from Sinopharm Chemical Reagent Co., Ltd methanol (≥99.9%) and acetonitrile (≥99.9%) for high-performance liquid chromatography (HPLC) were purchased from Sigma Aldrich. The purified water used in the experiment was obtained from a purification system with a resistivity of 18 MO cm^−1^. All other reagents and chemicals were of analytical grade and used without further purification.

Non-small cell lung cancer cells (A549), mouse monocyte macrophage leukemia cells (RAW264.7), human liver cancer cells (Hep G2), Human gastric mucosal epithelial cells (GES-1) and human normal hepatocytes (L02) were purchased from the Shanghai Cell Bank of the Chinese Academy of Sciences.

### 2.2 Synthesis of carbon dots

0.8408 g CA and 0.5224 g l-NAC (the molar ratio of CA to l-NAC is CA : l-NAC = 5 : 4) were dissolved in 16 mL water ultrasonically, and 6 parts of 2 mL solution were taken into the inner lining of the reactor after opening the lid, react for 12 h in an oven at 70 °C. After that, N_2_ was continuously injected into each lining for 3 min, sealed, and reacted in an oven at 200 °C for 0 h, 0.5 h, 1 h, 1.5 h, 2 h, and 2.5 h. After the reaction, methanol was ultrasonically dissolved several times and transferred to a 25 mL volumetric flask to a constant volume for HPLC analysis. At the same time, 0.8408 g CA and 0.9832 g GSH (CA : l-NAC molar ratio of 5 : 4) were dissolved in 16 mL of water ultrasonically and reacted under the same conditions as above for HPLC analysis.

18.3925 g CA and 11.4275 g l-NAC were dissolved in 350 mL of water ultrasonically. The second step was the reaction for 2.5 h at 200 °C, and the rest of the process was consistent with the above. The product was labeled as CDs-NAC for separation. 12.8222 g of CA and 14.8709 g of GSH were dissolved in 242 mL of water ultrasonically, similar to the synthesis process of CDs-NAC, and the product was labeled CDs-GSH for separation.

The obtained product was ultrafiltered through a 1000 mW ultrafiltration tube at 4000 rpm for 20 min. The filtered solution was the required reaction intermediate small molecule.

A small quantity of CDs were dialyzed with 500 Da dialysis bag. The purified product was lyophilized and observed by Transmission Electron Microscope(Talos F200X Thermo Scientific).

### 2.3 Separation of obtained products

After ultrafiltration and centrifugation, CDs-NAC (17.6 g) was separated by normal phase column chromatography column chromatography. After 100% dichloromethane, dichloromethane : methanol = 500 : 1, 100 : 1, 30 : 1, 9 components were obtained after gradient elution, respectively A-1-A-9, and the masses were 60.0 mg and 20.2 mg respectively. 23.9 mg, 1.0 mg, 14.4 mg, 21.7 mg, 39.4 mg, 18.2 mg, 15.7 g; A-3 RP-HPLC to obtain 3.5 mg blue-green (A-BG) and 1.5 mg green (AG) two single fluorescence Components. Elution conditions: 28%-28%-100%-100% acetonitrile, 0-40-45-60 min; A-8 dry method mixes the sample through normal phase column chromatography and passes through dichloromethane : methanol = 200 : 1 after elution, two components were obtained, namely A-8-1-A-8-2, with masses of 30.2 mg and 98.3 mg respectively; A-8-1 was dried and mixed with normal phase column chromatography, after 100% elution with dichloromethane, 10.0 mg of blue fluorescent component (AB).

After ultrafiltration and centrifugation,CDs-GSH (20.0 g) was mixed with the dry method and passed through normal phase column chromatography. After dichloromethane : petroleum ether = 1 : 1, dichloromethane : methanol = 100 : 1, 30 : 1, 6 components were obtained after gradient elution, respectively, are B-1-B-6, and the masses are 1.0 mg, 803.5 mg, 814.0 mg, 35.0 mg, 903.1 mg, 15.3 mg; B-2 dry-mixed samples are subjected to normal phase column chromatography, and then subjected to dichloromethane : methanol = 500 : 1, 3 components are obtained after elution, namely B-2-1-B-2-3, the masses are 1.0 mg, 20.0 mg, 689.2 mg respectively; B-2-2 passed RP-HPLC obtain 1.5 mg B-2-2-1, 1.0 mg blue-green fluorescent component (B-BG-1). Elution conditions: 28%-28%-100%-100% acetonitrile, 0-30-35-60 min; B-4 passed RP-HPLC to obtain two components. They are B-4-1-B-4-2, and the masses are 20.0 mg and 13.5 mg respectively. Elution conditions: 30%-30% acetonitrile, 0–20 min; B-4-2 dry mix sample and pass normal phase column chromatography, pass dichloromethane : methanol = 100 : 1, and obtain two components after elution. They are B-4-2-1-B-4-2-2, and the masses are 9.8 mg and 1.5 mg respectively; B-4-2-1 dry-mixed samples are subjected to normal phase column chromatography, and then petroleum ether: acetic acid ethyl ester = 7 : 3, 3 : 2, 1 : 1, 5 : 1 gradient elution to obtain 1.0 mg of blue-green fluorescent component (B-BG-2).

### 2.4 Characterization

Analysis of the synthetic products of CDs under different conditions was conducted on a 4.6 × 250 mm BDS HYPERSIL-C18 5 μm column (Thermo Scientific, USA) using an Ultimate-3000 HPLC system (Dionex, USA) equipped with an Ultimate-3000 diode arraydetector (DAD) and an Ultimate-3000 fluorescence detector. Separation of CDs was performed on a 10.0 × 250 mm YMC Pack ODS-A S-5 μm, 12 nm column (YMC Korea Co., Ltd). UV-visible absorption spectra were acquired using a Lambda-35 UV/visible spectrophotometer (PerkinElmer Company). Fluorescence spectra were recorded using an LS55 spectrofluorometer (PerkinElmer Company). Fourier transform-infrared spectra were obtained on a Nicolet 6700 (FT-IR) spectrometer (Thermo Fisher Scientific), and the sample mixed with potassium bromide powder was measured at room temperature. Hydrogen spectrum (^1^H-NMR) and carbon spectrum (^13^C-NMR) were recorded on a Bruker Ascend IIITM 600 MHz NMR spectrometer (Bruker). Electrospray ionization mass spectrometry (ESI-MS) was performed using a UHPLC system and a Q Exactive HF mass spectrometer (*Q Exactivee*Q Exactive).

### 2.5 QY calculations

The QYs of the CDs were calculated by comparing the integrated PL intensities and absorbance values of the samples (excited at 320 nm), using quinine dissolved in 0.1 mol L^−1^ H_2_SO_4_ aqueous solution(refractive index (*η*) = 1.33) as the standard (QY = 54.6%).^31^ All samples dissolved in water (*η* = 1.33) had an absorbance of less than 0.1 at 320 nm. The relative QY was calculated using the following equation:*Φ*_X_ = *Φ*_ST_ (Grad_X_/Grad_ST_)(*η*_X_^2^/*η*_ST_^2^)where *Φ* is the QY, Grad is the gradient from the plot of integrated fluorescence intensity *versus* absorbance, *η* is the refractive index of the solvent, ST is the standard, and X is the sample.

### 2.6 *Vitro* cytotoxicity-test (MTT method)

RAW264.7, L02, Hep G2, and A549 cells were inoculated into 96 well plates and incubated at 37 °C overnight. The cells were treated with 500 μg mL^−1^ of CDs-NAC and CDs-GSH for 24 h. After removing the supernatant, MTT was detected at 492 nm wavelength.

### 2.7 Cell imaging

L02 cells were incubated with 200 μg mL^−1^ of CDs-NAC and CDs-GSH in a carbon dioxide incubator for 3 h, the upper solution was discarded, PBS was washed three times, and cell staining was observed under a laser confocal microscope (FV1000 Olympus). GES-1 cells were treated in the same way. Dosing concentration is set to 100 μg mL^−1^, 200 μg mL^−1^, 300 μg mL^−1^.

## 3. Results and analysis

### 3.1 Separation result and structure characterization

**Fig. 1:**
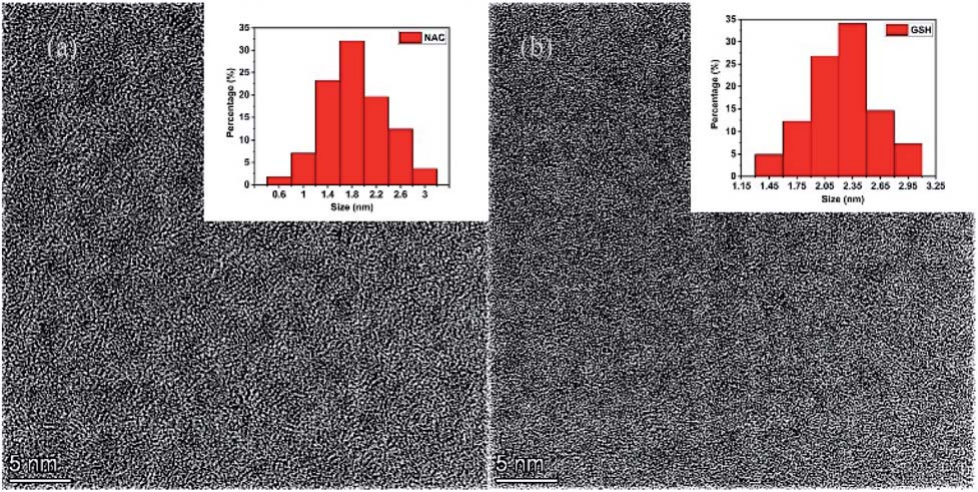
TEM images of CD-NAC (a) and CD-GSH (b). The insets are the particle size distribution pictures of CD-NAC and CD-GSH, respectively.

**Fig. 2:**
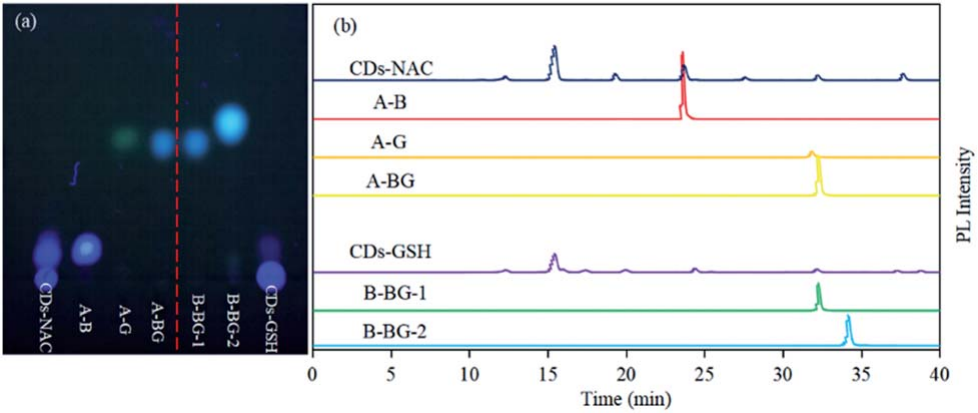
(a) The TLC results of CDs-NAC, CDs-GSH and all fluorophores separated from CDs-NAC and CDs-GSH. All of these samples were run under this condition of eluent (dichloromethane : methanol = 50 : 1). (b) Fluorescence chromatograms of CDs-NAC, CDs-GSH, and the separated fluorophores from CDs-NAC and CDs-GSH were monitored at 365 nm/440 nm. All analyses were carried out under the same HPLC conditions. Column temperature: 25 °C; eluent ratio and time: acetonitrile and water were used as two phases, and all samples were eluted with acetonitrile whose concentration ranged from 15% to 55% over 45 min.

In order to obtain monomeric fluorophores for better exploration on the impacts of different raw material carbon sources upon the formation mechanism and fluorescence mechanism of CDs, CA and two cysteine analogs (l-NAC and GSH) have been used as carbon sources to prepared CDs-NAC, and CDs-GSH by pyrolysis through two-step route. The raw materials were firstly heated in an oven at 70 °C for 12 h, and the products was then heated at 200 °C for 3 h under the premise of nitrogen as a protective gas. TEM was used to characterize the morphology and dispersion of the prepared CDs-NAC and CDs-GSH. The TEM images show that both CDs-NAC and CDs-GSH were in regular spherical shape with excellent dispersion (Fig. 1) and the particle distributions were relatively uniform (Fig. 1 inset). Since the macromolecular nanostructure CDs are difficult to be eluted by thin layer chromatography (TLC) and HPLC, but small molecule fluorophores can be easily eluted, as shown in the TLC images of CDs-NAC in Fig. 2a, CDs remain at the origin while the fluorophores have been spread a certain distance from the origin. The HPLC fluorescence spectrum (Fig. 2b) and the UV spectrum (Fig. S4†) of CDs-NAC show peaks of CDs in the liquid phase. When CDs-NAC were separated by column chromatography and HPLC (Fig. S3a†), three monomeric fluorophores (AB, AG and A-BG) were obtained, which display blue, blue-green and green fluorescence, as shown in Fig. S1a and c.† CDs-NAC TLC results (Fig. 2a), HPLC fluorescence spectrum (Fig. 2b), and ultraviolet spectrum (Fig. S4†). This is consistent with the blue luminescence of CDs-NAC body (Fig. 2b and d). The separation result of CDs-GSH was similar to that of CDs-NAC. When CDs-GSH was separated by column chromatography and HPLC (Fig. S3b†), two monomer fluorophores (B-BG-1 and B-BG-2) were obtained, as shown in Fig. S1a and c.† The TLC results (Fig. 2a), HPLC fluorescence spectrum (Fig. 2b) and ultraviolet spectrum (Fig. S4†) of CDs-GSH all indicate the existence of trace amount of blue-green fluorescent fluorophores, as in line with the main body of CDs-GSH. This is consistent with blue light (Fig. S1b and d†). It is worth mentioning that the positions of A-BG and -B-BG-1 were the same on TLC and HPLC, and they were found to be the same compound through structural identification, indicating that CDs-NAC and CDs-GSH, which are polymerized by fluorophores, may undergo a similar synthesis process.

**Fig. 3:**
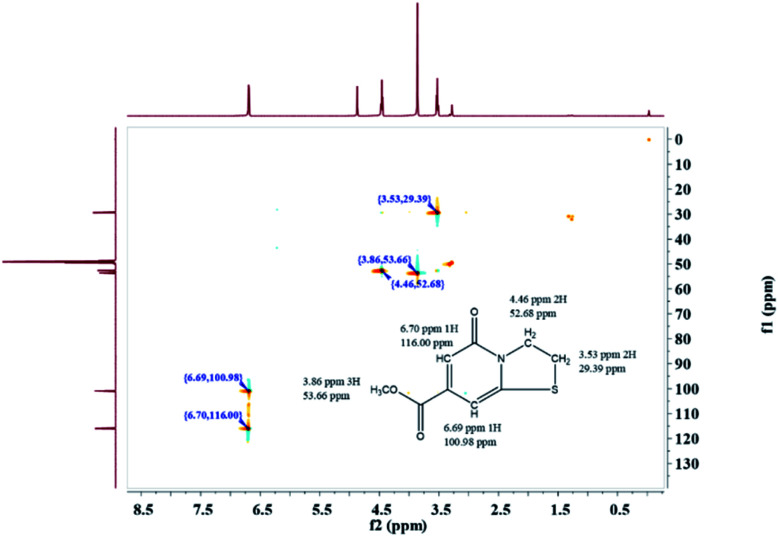
HSQC spectrum of A-B.

The precise characterization of the fluorophore structures would be of guiding significance for the derivation of the CDs formation mechanism and fluorescence mechanism. ^1^H-NMR, ^13^C-NMR, Dept135-NMR, HSQC-NMR, HMBC-NMR, and ESI-HRMS (Fig. S5–34†) were used to characterize the structures of the separated fluorophores. For example, the HSQC spectrum of A-B (Fig. 3) and fluorophore structure show a good correlation. All information related to the structure of the isolated fluorophores is presented in Table 1.

**Table 1:** Different color fluorescent compounds separated from CDs-NAC and CDs-GSH

Code	Compound structure	Chemical formula (ESI-HRMS)	Color
A-B	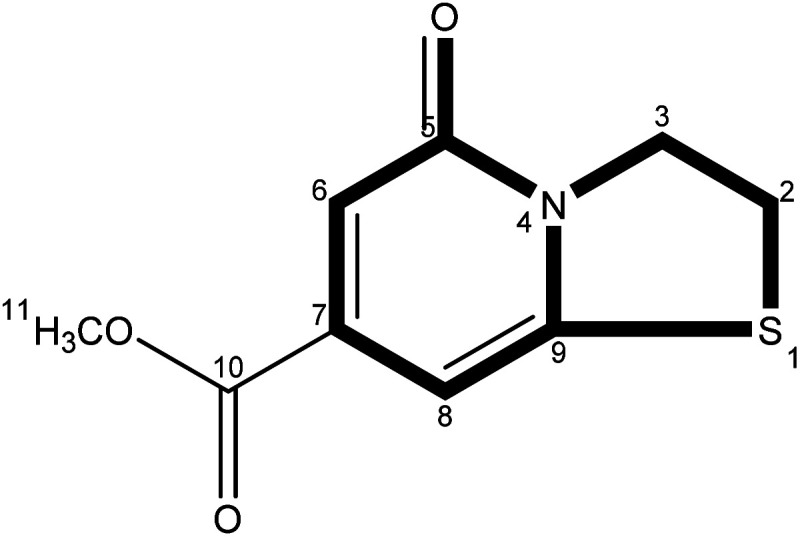	C_9_H_9_NO_3_S (212.0380)	Blue
A-G	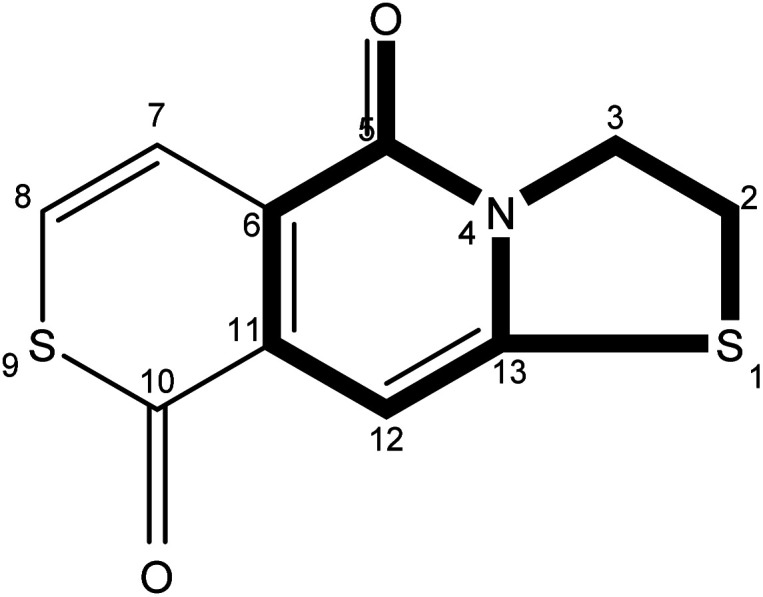	C_10_H_7_NO_2_S_2_ (237.9991)	Green
A-BG/B-BG-1	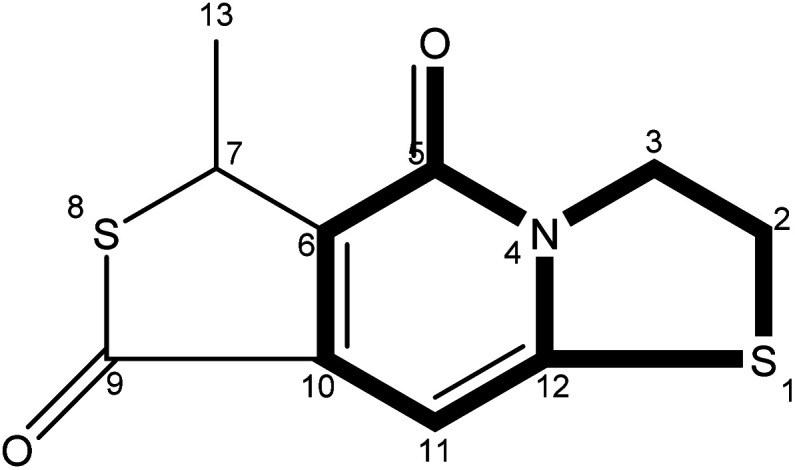	C_10_H_9_NO_2_S_2_ (240.0147)	Blue-green
B-BG-2	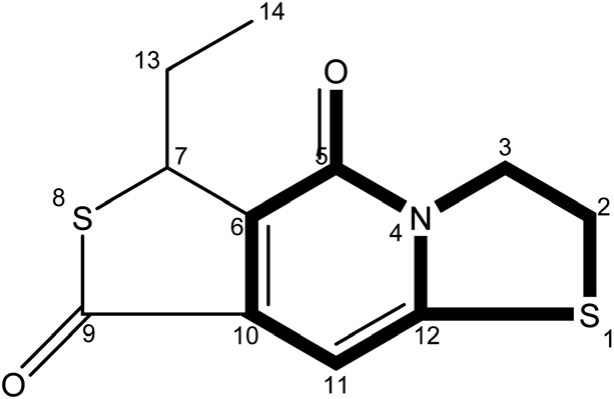	C_11_H_11_NO_2_S_2_ (254.0303)	Blue-green

The fluorescence properties of fluorophores stem from their structures, which would further affect the luminescence properties of prepared CDs. The fluorophores isolated from CDs-NAC and CDs-GSH all satisfy the basic structure of “3-dihydrothiazolo [3,2-a]pyridin-5-one”. The difference between the type of substituents on α-C and the carboxyl group on amide β-C dehydrated to ester is the main difference between the separated fluorophores (because there is no substituent on α-C of A–B amide, carboxyl groups on β-C of A–B amide are dehydrated into esters with external groups). When the substituent is “H”, the fluorophore emits blue fluorescence (*λ*_em_ = 441 nm); while the substituent is “–S–CH(CH_3_)–,” the fluorophore emits blue-green fluorescence (*λ*_em_ = 468 nm) which is attributed to the “n–π” transition caused by sulfur doping that leads to the emission wavelength shifting. When the substituent is “–S–CH=CH–,” the fluorescence of fluorophore is green (*λ*_em_ = 489 nm), which is attributed to the expansion of the conjugation system by the introduction of double bands, leading to a further red shift of emission wavelength. Though the emission wavelengths of “–S–CH(CH_3_)–” and “–S–HCH_2_(CH_3_)– ” are the same at 468 nm, the quantum yield of “–S–CH(CH_3_)–” is lower than that of “–S– HCH_2_(CH_3_)– ”. This indicates that the appropriate introduction of alkyl groups would not change the emission wavelength of fluorophore, but can enhance its fluorescence intensity. It is worth mentioning that the AG isolated from CDs-NAC and the B-BG-1 isolated from CDs-GSH are the same compound, showing that the synthesis of CDs with CA and different cysteine analogs as carbon sources is the similar formation processes.

### 3.2 The formation pathway of CDs-NAC and CDs-GSH

**Fig. 4:**
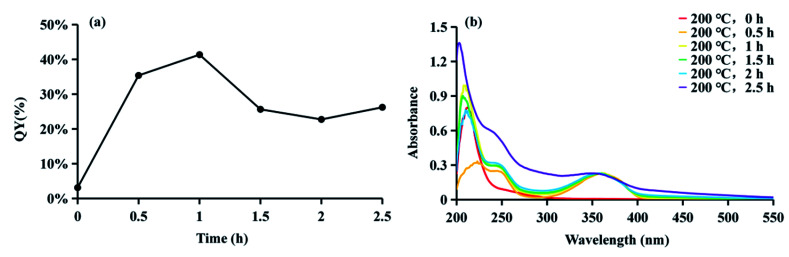
(a) The QYs and (b) UV-visible absorption spectra of CDs-NAC prepared at 200 °C for various reaction times in the second stage (0, 0.5, 1, 1.5, 2 and 2.5 h) (*λ*_ex_ = 365 nm).

**Fig. 5:**
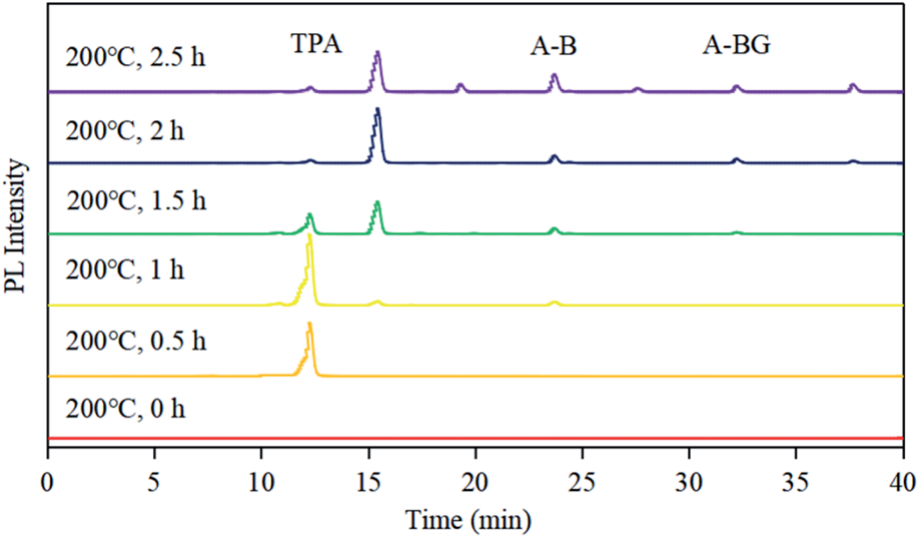
The fluorescence chromatograms of CDs-NAC at the different reaction time of the second step monitored at 365 nm/440 nm. All analyses were carried out under the same HPLC conditions. Column temperature: 25 °C; eluent ratio and acquisition time: acetonitrile and water were used as two phases, and all samples were eluted with acetonitrile whose concentration ranged from 15% to 55% over 45 min.

CDs is synthesized at two stages. The first step is mixing the raw materials CA and cysteine analogs (l-NAC and GSH) uniformly by water evaporation, and in this process, a small amount of fluorescent substances are generated. The second step mainly involves the large-scale synthesis of fluorophores and the formation of CDs by dehydration and polymerization at high temperatures. Unlike CDs, the fluorescence emission spectrum shows that the product of the first stage is not excitation-dependent (*λ*_ex_ = 365 nm, *λ*_em_ = 440 nm), and the QY is extremely low at 3.12% (Fig. 4a), showing pretty low reaction degree of fluorophore formation in the first stage, and the second stage is the main stage for CDs formation. To better understand the formation process of CDs, the QY, UV-Vis scan pattern and HPLC analysis results of prepared CDs-NAC in the second stage were recorded every 0.5 h (0 h, 0.5 h, 1 h, 1.5 h, 2 h, 2.5 h). In the first hour of the second stage, QYs of the product increases with reaction time, and reached maximum at 1 h. The UV-visible scan (Fig. 4b) shows a highlight peak at 365 nm derived from the “n–π” transition co-doped with nitrogen and sulphur, illustrating that during this period, the formation of nitrogen–sulfur co-doped fluorophore is the main synthesis reaction. Ds-NAC HPLC fluorescence detection results (Fig. 5) and UV detection results (Fig. S35a†) provided further proofs for this process. In the fluorescence chromatographic spectrum of the product prepared for 0.5 h, only a peak at 12.36 min appeared, while for the product prepared for 1 h, two new peaks appeared at 15.51 min and 23.90 min, and the fluorescence intensity reached maximum at 12.36 min peak. This change is consistent with the change in QYs of the products. The liquid chromatography mass spectrometry (LC-MS) proved that the peak at 12.36 min was estimated to be 5-oxo-2,3-dihydro-5*H*-[1,3] thiazolo[3,2-*a*]pyridine-3,7-dicarboxylic acid (TPA)27 (Fig. S37†). The above data indicates that in the first hour, the main reaction is the formation of fluorophore TPA.

**Scheme 1:**
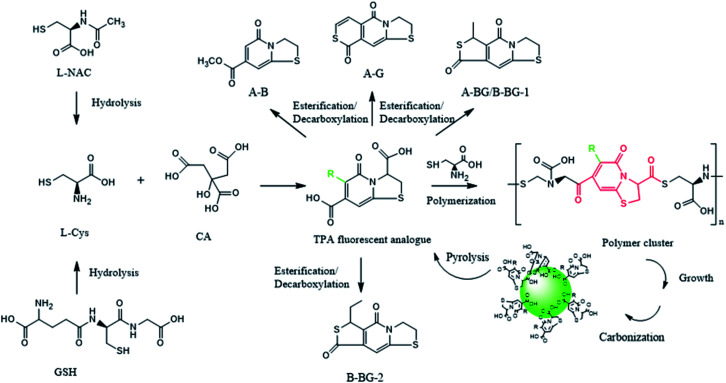
Proposed formation and pyrolysis pathway of carbon dots generated by CA + l-NAC or CA + GSH. Substituent R can be described as the following: H, –S–CH=CH–, –S–CH(CH_3_)– and –S–CHCH_2_(CH_3_)–.

When the reaction of the second stage keeps going for another 0.5 h, the QY of the product dropped sharply (Fig. 4a), indicating the large amount of consumption of fluorophores to form carbon nuclei. The HPLC fluorescence detection results (Fig. 5) and the UV detection results (Fig. S35a†) of CDs-NAC further showed that the TPA content decreased rapidly at this stage, with enhanced peaks at 15.51 min and 23.90 min. A new peak at 32.71 min appeared. The peaks at 23.90 min and 32.71 min have been separated and identified as A-B and A-BG, showing that TPA is conversing into fluorophores A-B and A-BG. It is thus speculated that in 1–1.5 h period, TPA first partly becomes different types of TPA analogs through further reactions. The instability of TPA and TPA analogs would lead to their own transformation. The dehydration and polymerization turn TPA and TPA analogs into a carbon skeleton, making fluorophore structurally stable (Scheme 1). When the reaction goes to 1.5–2 h period, QY of the product is still falling (Fig. 4a), but this downward trend slows down. Simultaneously, the HPLC fluorescence detection results (Fig. 5) and UV detection results (Fig. S35a†) of CDs-NAC showed that TPA was consumed to a minimum through the conversion reaction, and the remaining peaks increased to varying degrees. The above results indicate that in this period, TPA continues to transform into small-molecule fluorophores and is used for carbon chain extension.

With the reaction time goes to 2–2.5 h, QY of the product (Fig. 4a) rebounds, probably due to the fluorophores falling off from the CDs caused by prolonged high-temperature heating. The UV-visible scan (Fig. 4b) shows that the peak at 252 nm (derived from the aromatic sp^2^ domain or the “π–π” transition of the carbonyl group) is significantly enhanced, and the peak band near 365 nm becomes wider, indicating the continuous dehydration and carbonization of CDs, and more fluorophores and CDs are formed. The HPLC fluorescence detection results (Fig. 5) and the UV detection results (Fig. S35a†) of CDs-NAC show other fluorophores increase except for the peak at 15.51 min. In addition, the appearance of new peaks at 19.78 min and 28.12 min indicates the conversion of the peak (15.51 min) at this stage is the reason for the increase in the types of fluorophores and CDs.

The fluorescence emission spectrum of the product CDs-GSH (Fig. S36b†) of the second reaction stage were recorded every (0.5 h 0, 0.5, 1, 1.5, 2, and 2.5 h). The QY (Fig. S36c†), UV-Vis scan (Fig. S36d†) and HPLC (Fig. S35b and c†) analysis reveal that the synthesis process of CDs-GSH was basically the same as that of CDs-NAC. The first step is the synthesis of the fluorophore with a low degree of reaction, and the second stage involves four stages of TPA synthesis: fluorophore polymerization, carbon chain extension, and carbonization. Similar to CDs-GSH, the long-term high-temperature environment causes some fluorophores falling off the CDs. In contrast to CDs-NAC, since GSH contains three amino acid compounds, non-fluorescent substances are formed in the synthesis process, like the one at 8.86 min (Fig. S35b and c†). By analysing the formation process of CDs-NAC and CDs-GSH, it is discovered that the essence of CDs synthesis with CA and cysteine analogs as carbon sources is actually the formation of l-cysteine by the hydrolysis of CA and cysteine analogs, as shown in Scheme 1. More importantly, the amide α-C of TPA generated during the reaction can be converted into diverse fluorophores (TPA/TPA analogs) by connecting different groups in the reaction, which then are dehydrated and polymerized into different carbon dots. This is of great significance for the design of CDs with richer fluorescent color when using CA and systeine as carbon sources.

### 3.3 Investigation of fluorescence emission sites and excitation dependence/independence

**Fig. 6:**
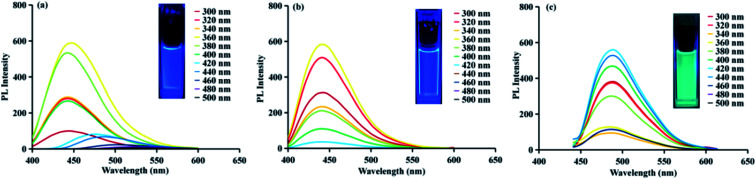
Fluorescence emission spectra of (a) CDs-NAC, (b) A-B and (c) A-G. The excitation wavelength of the fluorescence emission spectrum was determined to be from 300 to 500 nm and measured every 20 nm.

From the studies of the synthesis process, the fluorescence of prepared CDs originates from both the surface state (caused by the interaction of the nitrogen–sulfur co-doped structure and the carbon skeleton) and the fluorescent molecules bound to the inside and outside of CDs. Fluorophores are left over from the later stage of CDs synthesis process because these fluorophores do not participate in polymerization reaction, or the long-time heating makes fluorophores falling off from CDs. To better understand the source of CDs fluorescence, the fluorescence emission spectra of the CDs and separated fluorophores are recorded. As shown in Fig. 6c, for CDs-NAC, when the excitation wavelength shifts from 300 nm to 400 nm, the best emission wavelength stays at the same position (*λ*_em_ = 440 nm), but the fluorescence intensity increases with the excitation wavelength. That is, there is excitation-independent dependence, which is attributed to the surface state caused by nitrogen and sulfur co-doping. When the excitation wavelength changes from 400 nm to 500 nm, the optimal emission wavelength exhibits an obvious red shift, that is, CDs exhibit excitation dependence property which is attributed to the multiple molecular states of different fluorophores. The fluorescence spectra of A-B, A-BG and A-G separated from CDs show the same phenomenon. When the excitation wavelength changes from 300 nm to 500 nm, these fluorophores A-B (*λ*_em_ = 441 nm), A-G (*λ*_em_ = 489 nm), and A-BG (*λ*_em_ = 468 nm) all have only one specific emission peak, and their fluorescence are excitation independent, revealing the monomer fluorophores contain only one monocular state for fluorescence emission. Since CDs exhibit excitation dependence property in longer wavelength range (400–500 nm), the long-wavelength emitting fluorophores, such as A-G, are the main reason for this property.

CDs-GSH (Fig. S40d†), like CDs-NAC, exhibit the same optical properties. When the excitation wavelength shifts from 300–400 nm, the excitation-wavelength dependence is observed, while the excitation wavelength further shifts to 500 nm, it exhibits excitation dependence. Similarly, the B-BG-1 (Fig. S40b†) and B-BG-2 (Fig. S40c†) isolated from CDs-GSH both exhibit excitation dependence property, showing that in the bottom-up synthesis route, the luminescence of CDs is highly dependent on molecular residues or organic molecular fluorophores due to the incomplete reaction of precursor molecules. Fluorophores can exist on the surface of prepared CDs or disperse in the solution as free molecules.

### 3.4 Biocompatibility evaluation

**Fig. 7:**
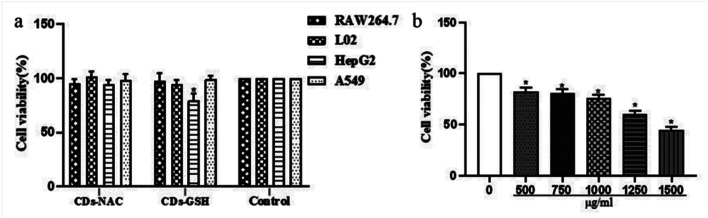
(a) Comparing the cell activity of each group, *n* = 3, * indicates that there is a significant difference compared with the blank group (*P* < 0.05). (b) Comparison of Hep G2 cell activity under different concentrations of CDs NAC, *n* = 3, * indicates that there is a significant difference compared with the blank group (*P* < 0.05).

Preliminary screening of CDs-NAC and CDs-GSH toxicity to cells is shown in Fig. 7a. 500 μg mL^−1^ CDs-NAC do not show any impact upon the viability of these four cell types, while for CDs-GSH at the same concentration, it is only toxic to Hep G2 cells. The CDs-GSH concentration was gradually increased from 500 μg mL^−1^ to 1500 μg mL^−1^ to calculate the IC50 of CDs-GSH on Hep G2 cells. As is shown in Fig. 7b, the toxicity of CDs-GSH to Hep G2 cells significantly enhances with the increase of CDs concentration, and the calculated IC50 of CDs-GSH in Hep G2 cells is 1499 ± 104 (*x* ± SD). The experiment results show good biocompatibility of both CDs, and the toxicity of CDs-GSH to Hep G2 cells can be further used in the field of anticancer drugs.

### 3.5 Cell imaging

**Fig. 8:**
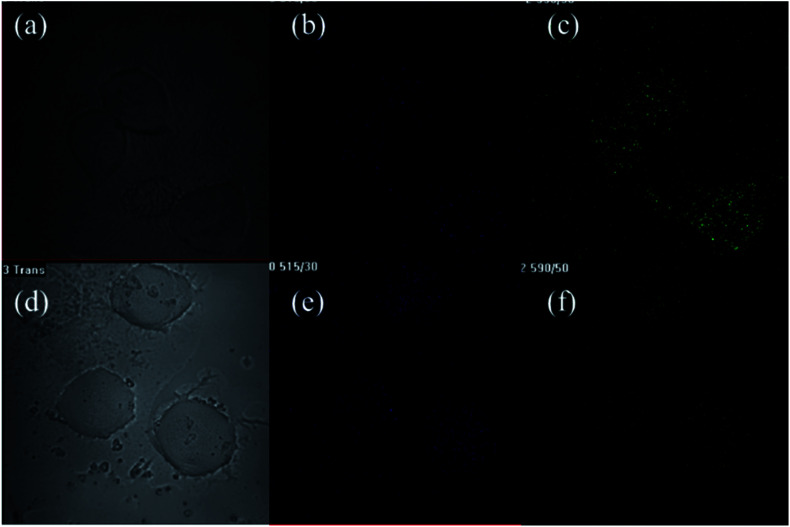
Cell imaging under laser microscope after incubation of CDs-NAC and CDs-GSH with L02 cells. (a–c) after incubation of CDs-NAC with L02 cells, images were taken at bright field, *λ*_ex/em_ = 408/515 nm and *λ*_ex/em_ = 488/590 nm, respectively. (d–f) After incubation of CDs-GSH with L02 cells, images were taken at bright field, *λ*_ex/em_ = 408/515 nm and *λ*_ex/em_ = 488/590 nm, respectively.

As shown in Fig. 8, after incubation with L02 cells, CDs-NAC and CDs-GSH emit blue and green fluorescence with *λ*_ex/em_ = 408/515 nm and *λ*_ex/em_ = 488/590 nm, respectively. The images show that CDs are mainly distributed in the cytoplasmic region of cells, and stain the cytoplasm but stay outside of the nucleus. Another cell GES-1 is also used to further demonstrate the cell imaging ability of prepared CDs (Fig. S41–S43†). Both CDs could exhibit cell shapes with blue and green fluorescence (*λ*_ex/em_ = 408/515 nm and *λ*_ex/em_ = 488/590 nm, respectively), and CDs are also mainly distributed in cytoplasm of the cells as the situation for LO2 cells. The results suggest that CDs can easily penetrate the cell membrane and label the cytoplasm of cells.

## 4. Conclusion

CDs-NAC and CDs-GSH have been synthesized by pyrolysis with CA and cysteine analogs (l-NAC and GSH) as carbon sources, blue, blue-green, and green fluorophores are separated by column chromatography and HPLC. The structure of the fluorophore was accurately characterized by NMR and ESI-HRMS, and the analysis show that the appropriate introduction of alkyl groups into the fluorophore structure of CDs can increase the fluorescence intensity. By combining HPLC analysis, ultraviolet-visible detection, fluorescence detection, and calculation of quantum yield, the formation process of CDs is deduced. Finally, it is found that the formation processes of CDs-NAC and CDs-GSH are basically the same, and both synthesis processes are essentially the reaction of CA and l-cys. The formation of CDs all undergo four stages of TPA synthesis: TPA analog polymerization, carbon chain extension, and carbonization. More importantly, during the reaction, the amide α-C of TPA can be converted into diverse fluorophores (TPA analogs) by connecting different groups through the reaction, and different fluorophores can be dehydrated and polymerized into different carbon dots. The discovery would be of great significance to design CDs with richer fluorescence colors. Through studying the excitation-dependent and non-excitation-dependent property of the separated fluorophores and the prepared CDs, CDs-NAC and CDs-GSH have non-excitation dependence under excitation at 300–400 nm, indicating that the CDs form the surface state caused by nitrogen–sulfur co-doping. The excitation dependence is observed at 400–500 nm, which reveals that the molecular state induced by various fluorophores is one of the reasons for the excitation dependence of CDs.

## Conflicts of interest

There are no conflicts to declare.

## Supplementary Material

RA-012-D2RA00431C-s001
