# Supplementary material for: The fluorescence mechanism of carbon dots based on the separation and identification of small molecular fluorophores
Source: RSC Adv. 2022 Apr 14;12(19):11640–8. doi: 10.1039/d2ra00431c (PMC9008442; doi:10.1039/d2ra00431c)
Supplement: RA-012-D2RA00431C-s001 [file RA-012-D2RA00431C-s001.pdf]

Electronic Supplementary Material (ESI) for RSC Advances.
This journal is © The Royal Society of Chemistry 2022

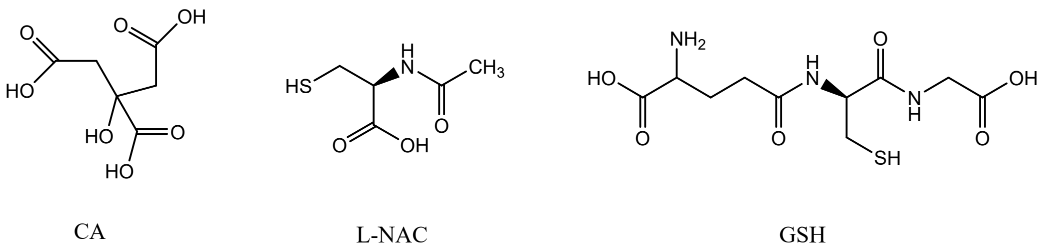

Fig. S.1. Structural formula of raw material of CDs-NAC and CDs-GSH containing CA, L-NAC and GSH.

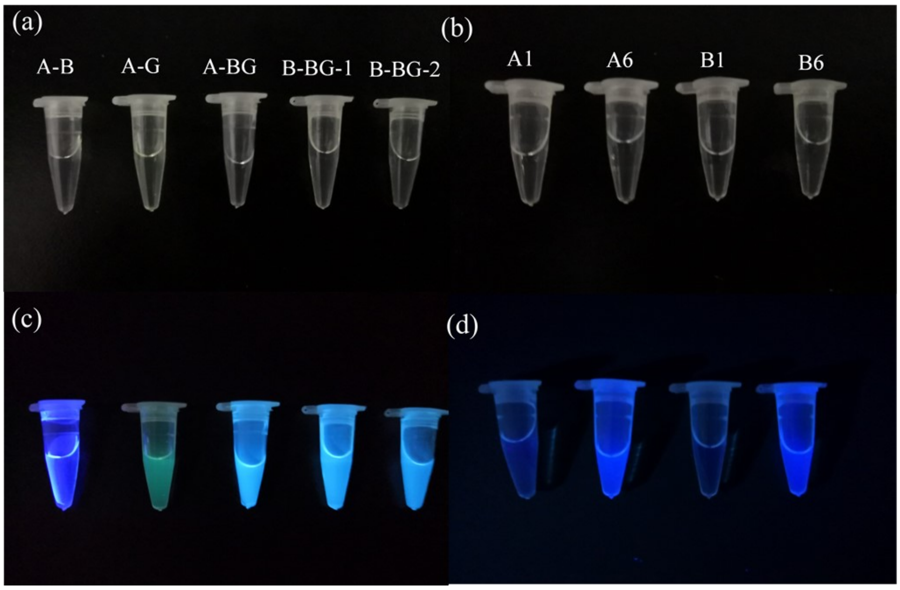

Fig. S.2. (a) Pictures of the separated fluorophores from CDs-NAC and CDs-GSH under white light. (b) Pictures of the first stage and final product of CDs-NAC and CDs-GSH under white light. (c) Pictures of the separated fluorophores from CDs-NAC and CDs-GSH under white 365 nm UV lamplight. (d) Pictures of the first stage and final product of CDs-NAC and CDs-GSH under 365 nm UV lamp.

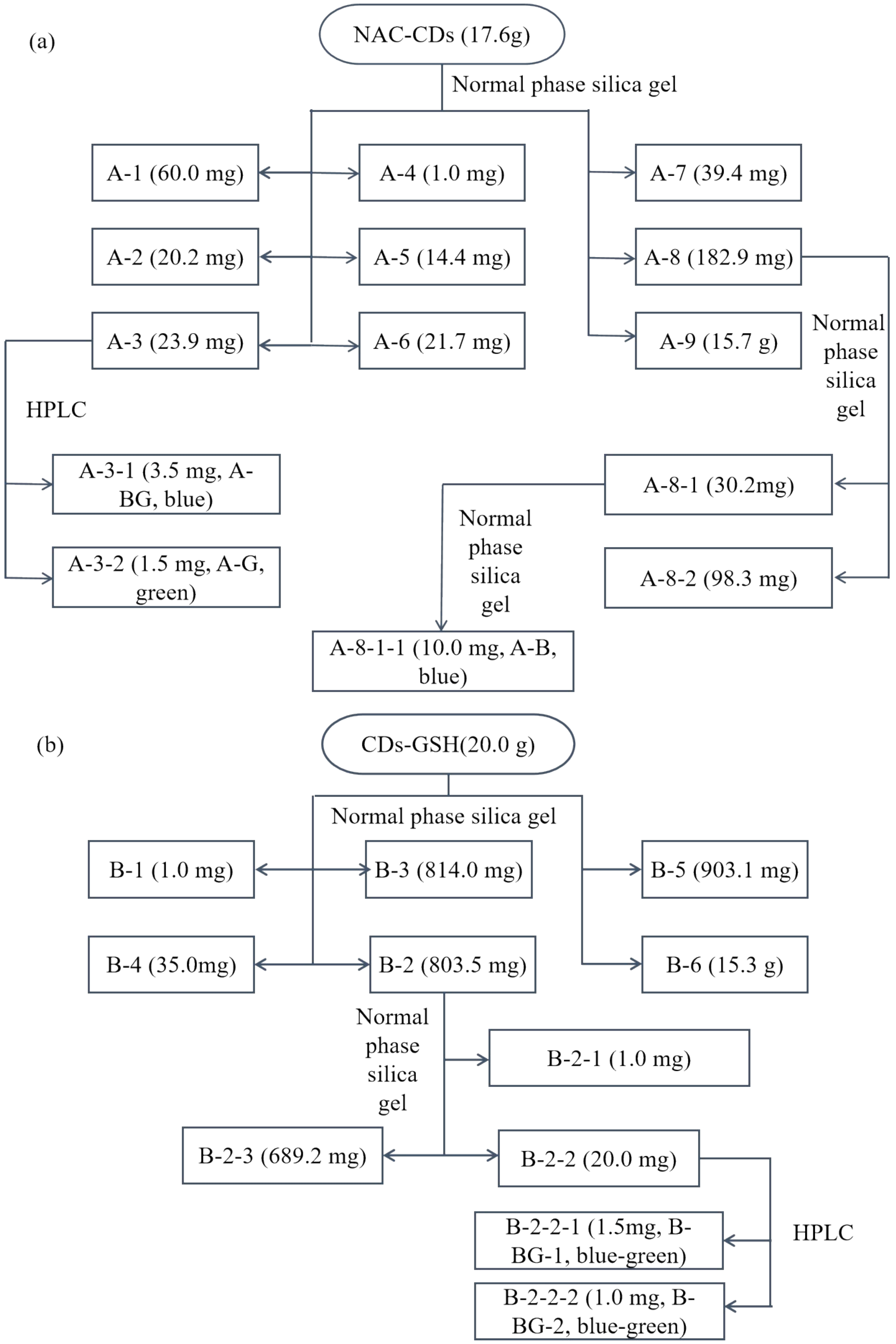

Fig. S.3. Flow chart of separation of (a) CDs-NAC and (b) CDs-GSH.

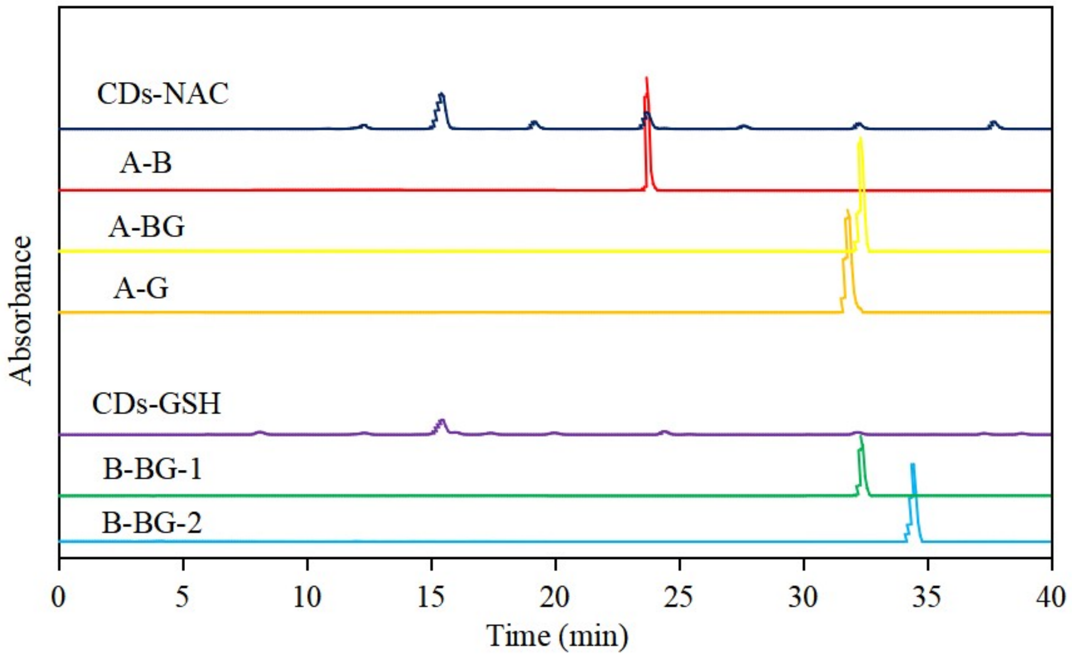

Fig. S.4. the absorption chromatograms of CDs-NAC, CDs-GSH and the separated fluorophores from CDs-NAC and CDs-GSH monitored at 365 nm. All of these were carried out under the same HPLC conditions. Column temperature: 25 °C; Eluent ratio and time: acetonitrile and water were used as two phases and all samples were eluted with acetonitrile whose concentration is from 15% to 55% during 45 min.

The Concrete results of NMR and ESI-HRMS of fluorophores separated from CDs-NAC and CDs-GSH.

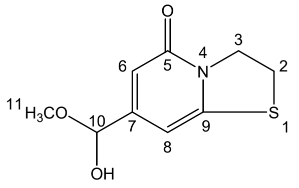

5-*oxo*-2,3-dihydrothiazolo[3,2-a]pyridine-7-carboxylate (A-B). Colorless amorphous powder. ^{1}H-NMR (600 MHz, CD_3DH-d_4): δ 6.69, 6.70 (2H, d), 4.46 (2H, t, J=7.2 Hz), 3.86 (3H, s), 3.53 (2H, t, J=7.2 Hz) ; ^{13}C-NMR (150 MHz, CD_3DH-d_4): δ 166.2 (C-10), 164.1 (C-5),152.5 (C-9), 143.5 (C-7), 116.0 (C-6), 100.9 (C-8), 53.6 (C-11), 52.6 (C-3), 29.40 (C-2), ; HRMS (ESI) $[M+H]^+$ $C_9H_9H^+NO_3S$ calcd 212.0376, found 212.0380 (Fig. S.5~S.10).

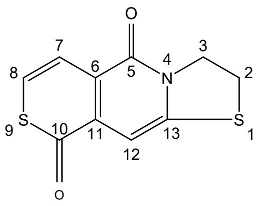

2,3-dihydrothiazolo[3,2-a]thiopyrano[3,4-d] pyridine -5,10-dione (A-G).

Green amorphous powder. ^{1}H-NMR (600 MHz, CD_3CL-d_3): δ 7.66 (1H, d, 7.2), 7.17 (1H, d, 7.2), 6.86 (1H, s,), 4.54 (2H, t, 7.2), 3.46 (2H, t, 7.2);

^{13}C-NMR (600 MHz, CD_3CL-d_3): 185.2 (C-10), 161.2 (C-5), 147.0 (C-13), 135.0 (C-11), 127.3 (C-6), 126.3 (C-8), 116.9 (C-7), 94.6 (C-12), 51.3 (C-3), 28.6 (C-2); HRMS (ESI) $[M+H]^+$ $C_{10}H_7H^+NO_2S_2$ calcd 237.9991, found 237.9991 (Fig. S.11~S.16).

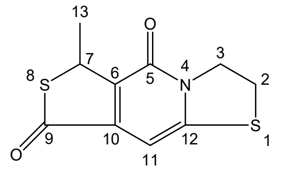

7-methyl-2,3-dihydrothiazolo[3,2-a]-7*H*-thieno[3,4-d]pyridine-5,9-dione (A-BG).

Light blue amorphous powder. ^{1}H-NMR (600 MHz, CD_3CL-d_3): δ 6.415 (1H, s), 4.768 (1H, q, J=7.2 Hz), 4.500 (2H, m, J_1=7.2 Hz, J_2=13.2 Hz), 3.48 (2H, t, J=7.2 Hz), 1.78 (3H, d, J=7.2 Hz); ^{13}C-NMR (600 MHz, CD_3CL-d_3): δ 196.1 (C-9), 159.1 (C-5), 150.1 (C-12), 145.8 (C-10), 138.5 (C-6). 93.5 (C-11), 50.7 (C-3), 44.9 (C-7), 29.4 (C-2), 19.8 (C-13); HRMS (ESI) $[M+H]^+$ $C_{10}H_9H^+NO_2S_2$ calcd 240.0148, found 240.0147 (Fig. S.17~S.22).

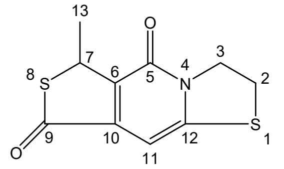

7-methyl-2,3-dihydrothiazolo[3,2-a]-7*H*-thieno[3,4-d]pyridine-5,9-dione (B-BG-1).

Light blue amorphous powder. ^{1}H-NMR (600 MHz, CD_3CL-d_3): δ 6.42 (1H, s), 4.77 (1H, q, J=7.2 Hz), 4.50 (2H, m, J_1=7.2 Hz, J_2=13.2 Hz), 3.49 (2H, t, J=7.2 Hz), 1.79 (3H, d, J=7.2 Hz); ^{13}C-NMR (600 MHz, CD_3CL-d_3): δ 196.1 (C-9), 159.1 (C-5), 150.1 (C-12), 145.8 (C-10), 138.5 (C-6). 93.5 (C-11), 50.7 (C-3), 44.9 (C-7), 29.4 (C-2), 19.8 (C-13); HRMS (ESI) $[M+H]^+$ $C_{10}H_9H^+NO_2S_2$ calcd 240.0148, found 240.0147 (Fig. S.23~S.28).

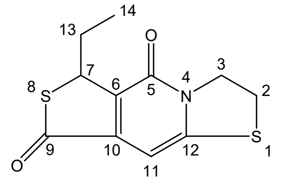

7-ethyl-2,3-dihydrothiazolo[3,2-a]-7*H*-thieno[3,4-d]pyridine-5,9-dione (B-G-2).

Light blue amorphous powder. ^{1}H-NMR (600 MHz, CD_3CL-d_3): δ 6.42 (1H, s), 4.74 (1H, d, J=9 Hz), 4.51 (2H, m, J_1=7.2 Hz, J_2=13.8 Hz), 3.48 (2H, t, J=7.2 Hz), 2.59, 1.82 (2H, m), 1.00 (3H, d, J=7.2 Hz); ^{13}C-NMR (600 MHz, CD_3CL-d_3): δ 196.4 (C-9), 159.1 (C-5), 150.0 (C-12), 146.2 (C-10), 137.3 (C-6). 93.6 (C-11), 52.5 (C-3), 50.7 (C-7), 29.3 (C-2), 26.3 (C-13) 12.1 (C-14); HRMS (ESI) $[M+H]^+$ $C_{11}H_{11}H^+NO_2S_2$ calcd 254.0304, found 254.0303 (Fig. S.29-S.34).

Table 1. The data about ^{1}H-NMR and ^{13}C-NMR of all fluorophores separated from CDs-NAC and

CDs-GSH.

Position	A-B		A-G		A-BG		B-BG-1		B-BG-2	
	^{1}H	^{13}C	^{1}H	^{13}C	^{1}H	^{13}C	^{1}H	^{13}C	^{1}H	^{13}C
2	3.53	29.4	3.46	28.6	3.48	29.4	3.49	29.4	3.48	29.3
3	4.46	52.6	4.54	51.3	4.50	50.7	4.50	50.7	4.51	50.5
5	—	164.1	—	161.2	—	159.1	—	159.1	—	159.1
6	6.69	116.0	—	127.3	—	138.5	—	138.5	—	137.3
7	—	143.5	7.66	116.9	4.77	44.9	4.77	44.9	4.74	50.7
8	6.70	100.9	7.17	126.3	—	—	—	—	—	—
9	—	152.5	—	—	—	196.1	—	196.1	—	196.4
10	—	166.2	—	185.2	—	145.6	—	145.8	—	146.2
11	3.86	53.6	—	135.0	6.42	93.5	6.42	93.5	6.42	93.6
12	—	—	6.86	94.6	—	150.1	—	150.1	—	150.0
13	—	—	—	147.0	1.78	19.8	1.79	19.8	1.82, 2.59	26.3
14	—	—	—	—	—	—	—	—	1.00	12.1

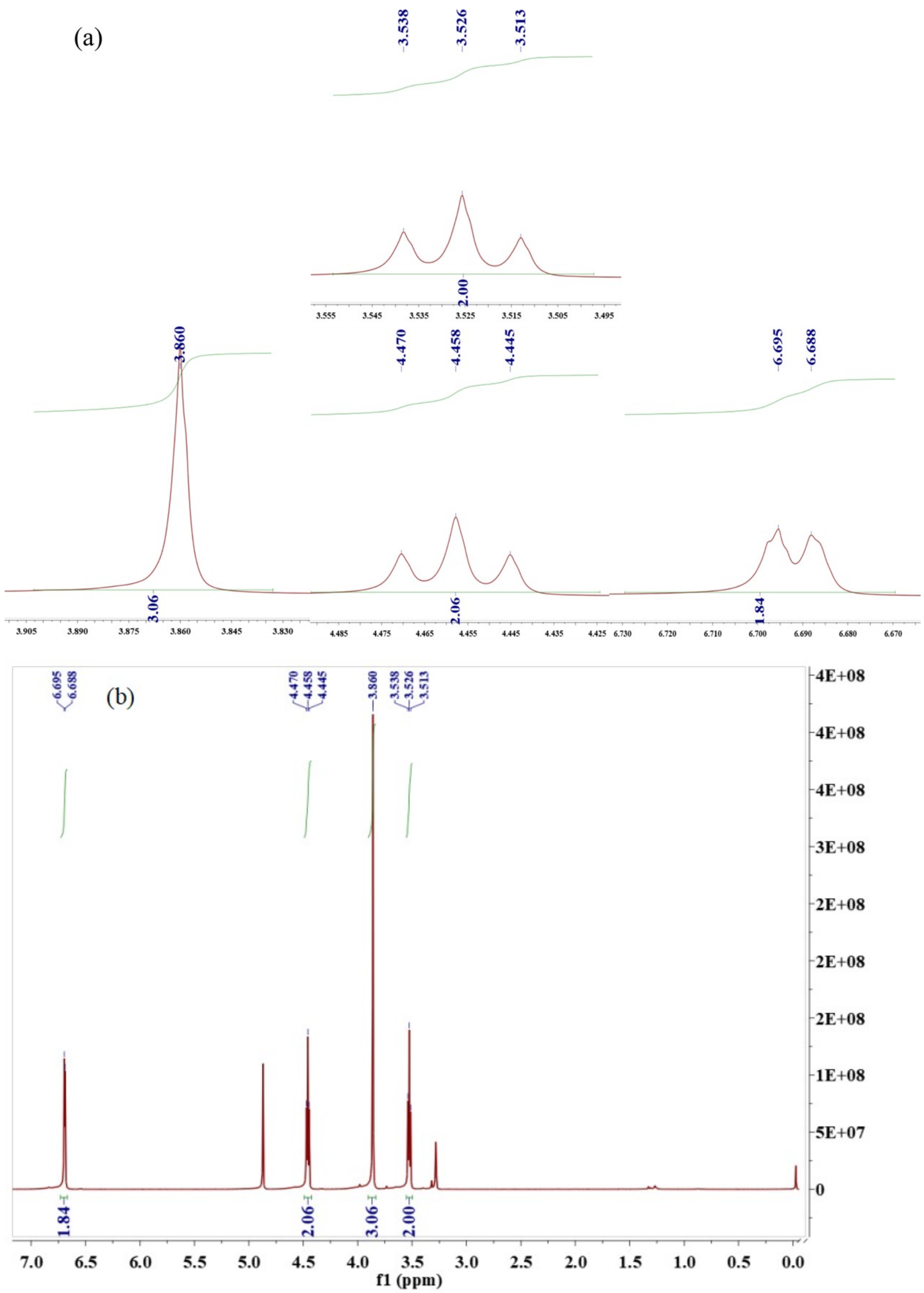

Fig. S.5. (a) the amplified spectrum of each peak at the ^{1}H-NMR spectrum. (b) ^{1}H-NMR spectrum of 5-oxo-2,3-dihydrothiazolo[3,2-a]pyridine-7-carboxylate (A-B) dissolved in CD_3OD.

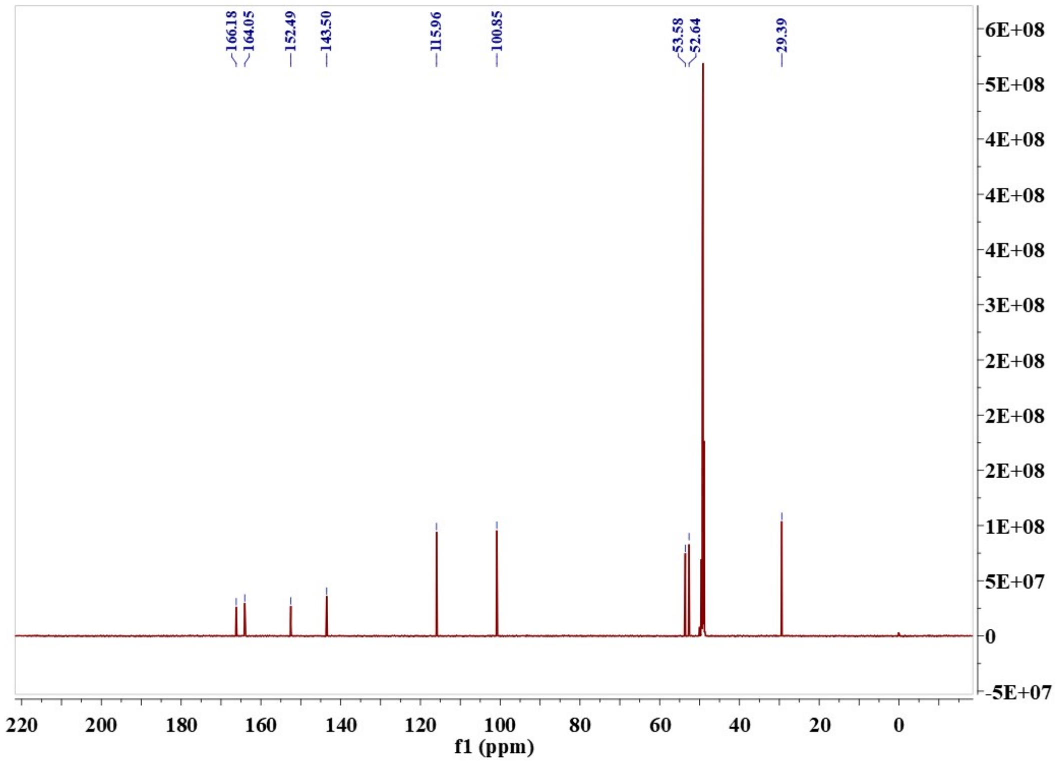

Fig. S.6. ^{13}C-NMR spectrum of 5-oxo-2,3-dihydrothiazolo[3,2-a]pyridine-7-carboxylate (A-B) dissolved in CD_3OD.

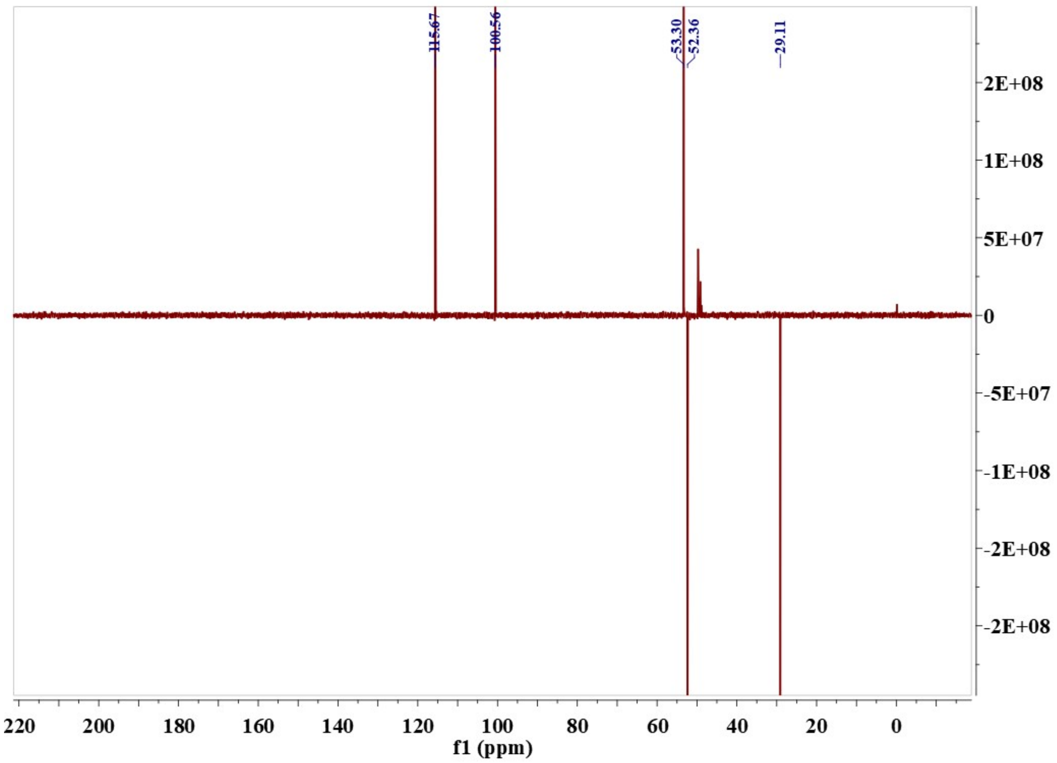

Fig. S.7. Dept 135-NMR spectrum of 5-oxo-2,3-dihydrothiazolo[3,2-a]pyridine-7-carboxylate (A-B) dissolved in CD_3OD.

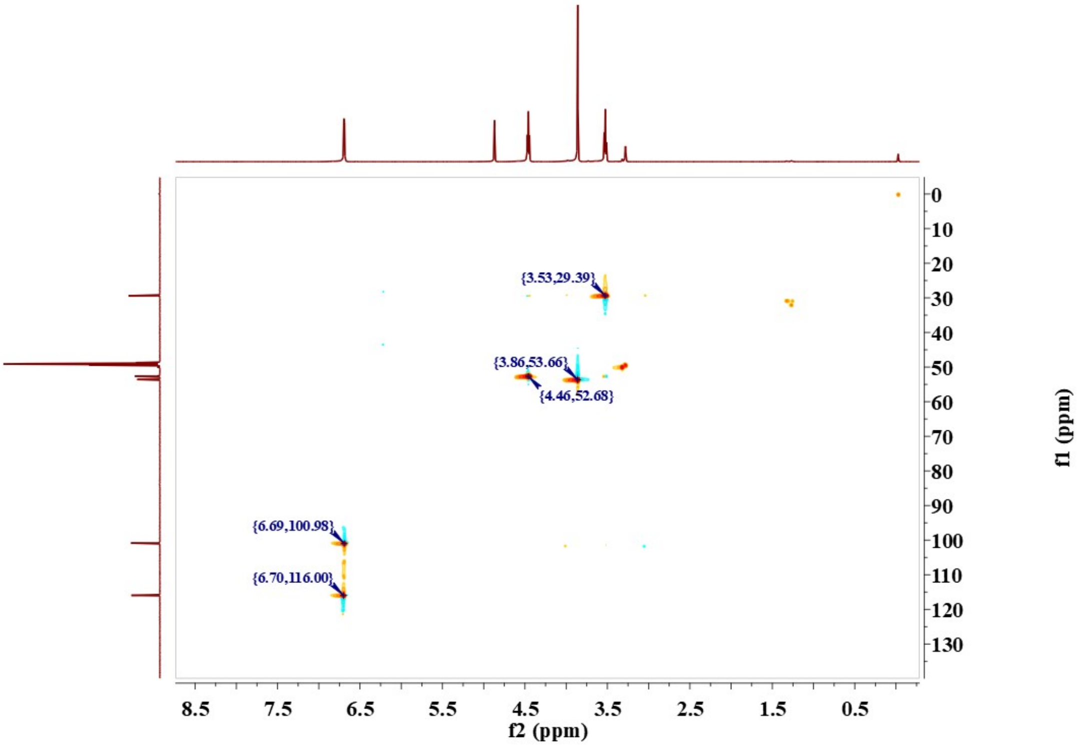

Fig. S.8. HSQC-NMR spectrum of 5-oxo-2,3-dihydrothiazolo[3,2-a]pyridine-7-carboxylate (A-B) dissolved in CD_3OD.

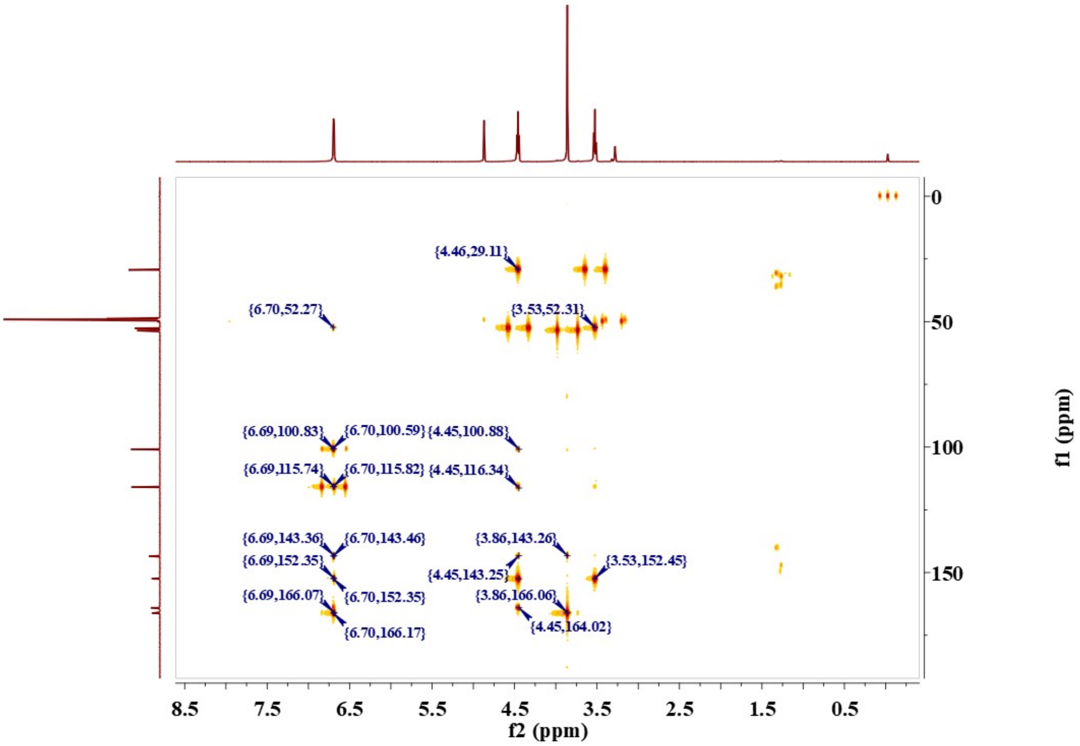

Fig. S.9. HMBC-NMR spectrum of 5-oxo-2,3-dihydrothiazolo[3,2-a]pyridine-7-carboxylate (A-B) dissolved in CD_3OD.

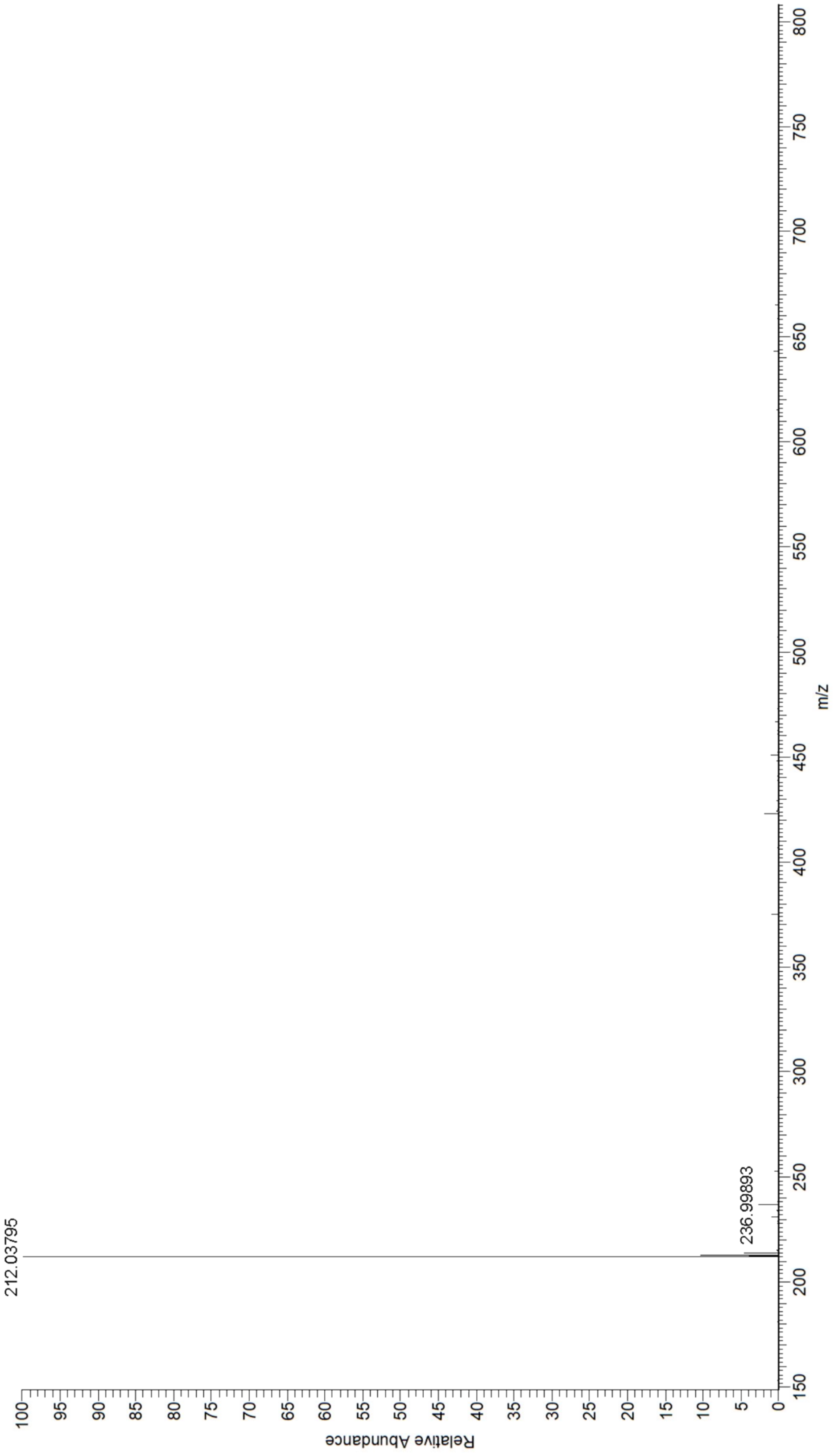

Fig. S.10. ESI-HRMS mass spectrum of methyl 5-oxo-2,3-dihydrothiazolo[3,2-a] pyridine-7-Carboxylate (A-B).

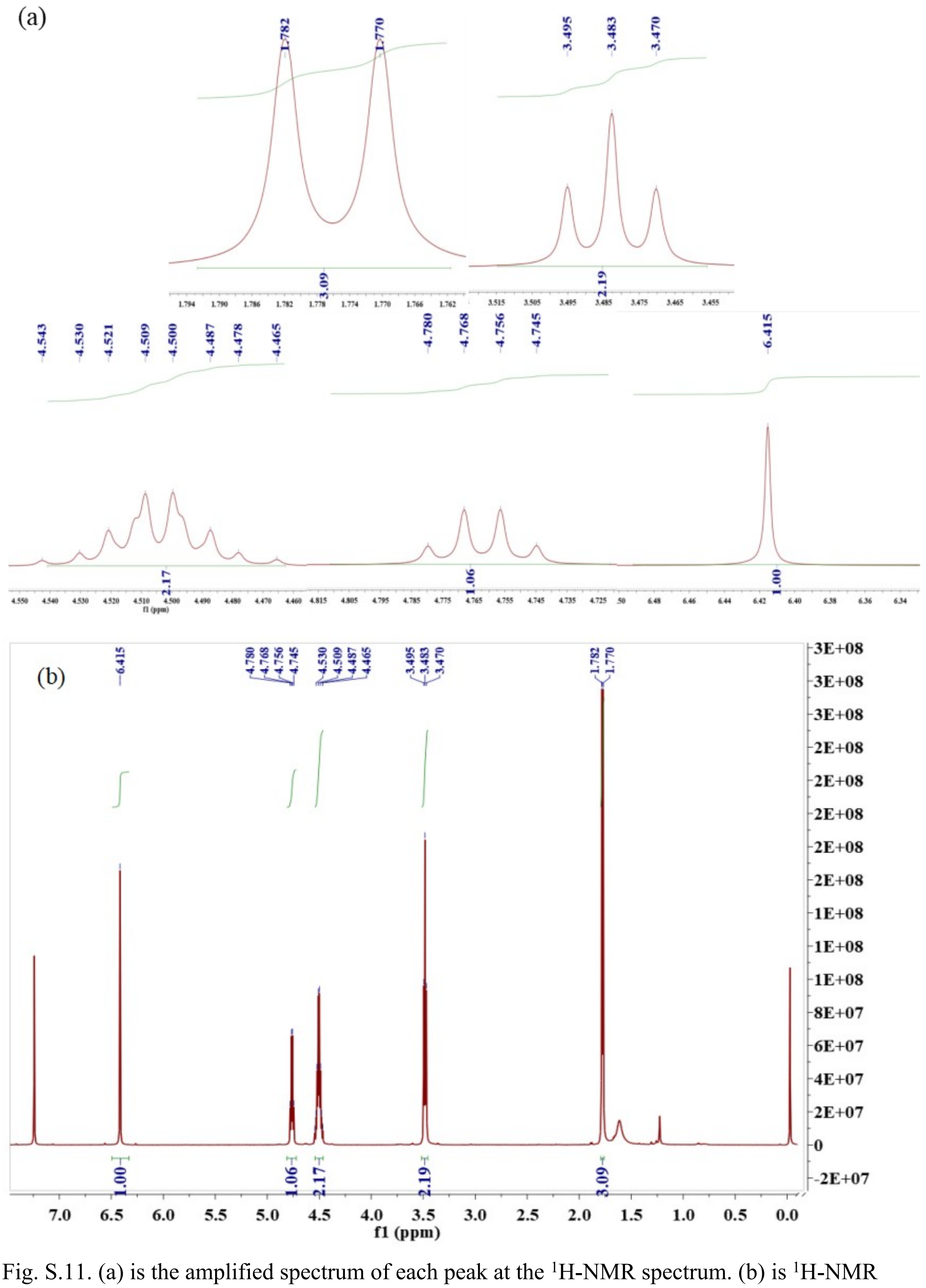

Fig. S.11. (a) is the amplified spectrum of each peak at the ^{1}H-NMR spectrum. (b) is ^{1}H-NMR spectrum of 2,3-dihydrothiazolo[3,2-a]thiopyrano[3,4-d]pyridine-5,10-dione (A-G) dissolved in CD_3CL.

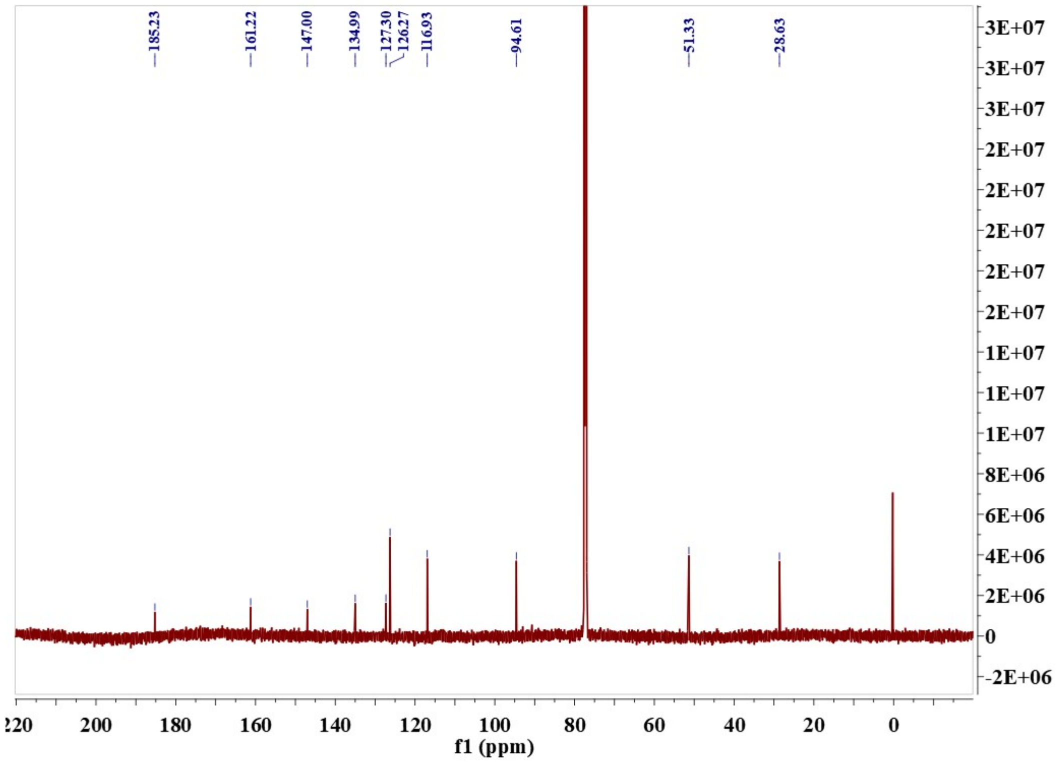

Fig. S.12. ^{13}C-NMR spectrum of 2,3-dihydrothiazolo[3,2-a]thiopyrano[3,4-d]pyridine-5,10 -dione (A-G) dissolved in CD_3CL.

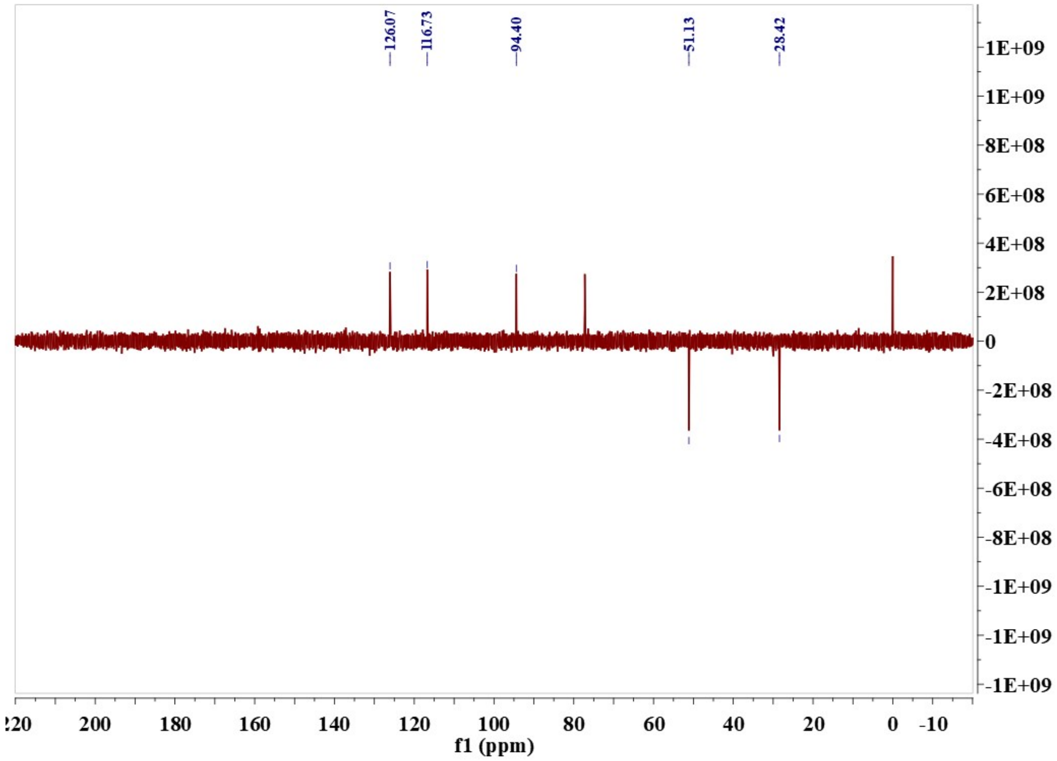

Fig. S.13. Dept 135-NMR spectrum of 2,3-dihydrothiazolo[3,2-a]thiopyrano[3,4-d] pyridine-5,10-dione (A-G) dissolved in CD_3CL.

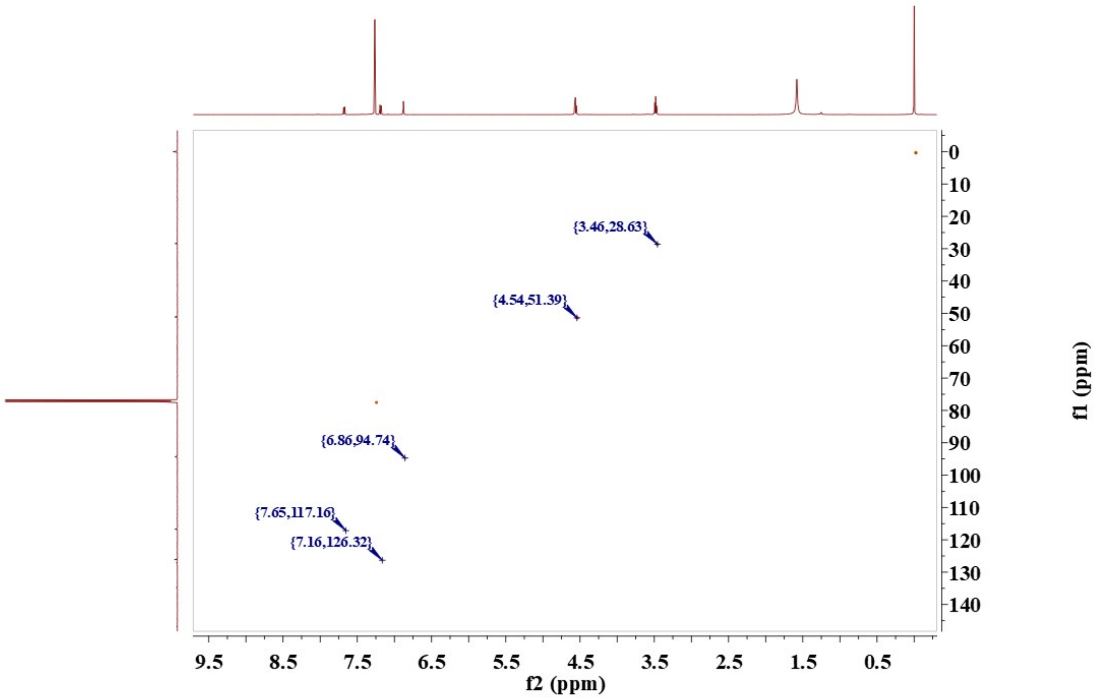

Fig. S.14. HSQC-NMR spectrum of 2,3-dihydrothiazolo[3,2-a]thiopyrano[3,4-d] pyridine -5,10-dione (A-G) dissolved in CD_3CL.

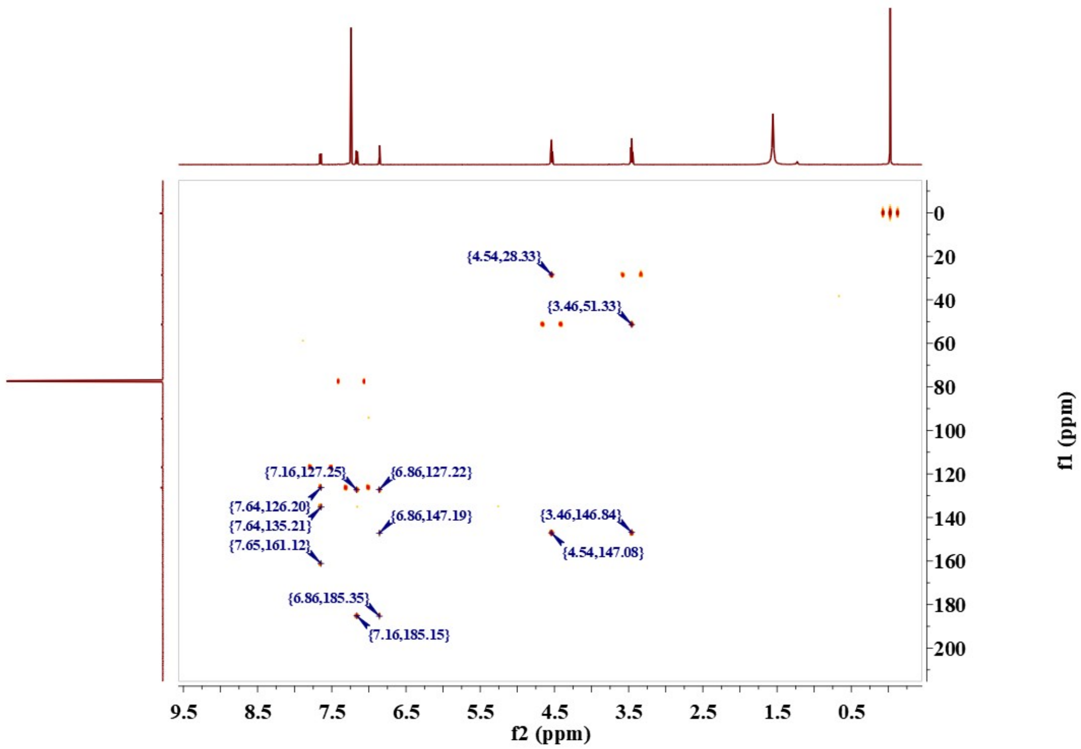

Fig. S.15. HMBC-NMR spectrum of 2,3-dihydrothiazolo[3,2-a]thiopyrano[3,4-d] pyridine -5,10-dione (A-G) dissolved in CD_3CL.

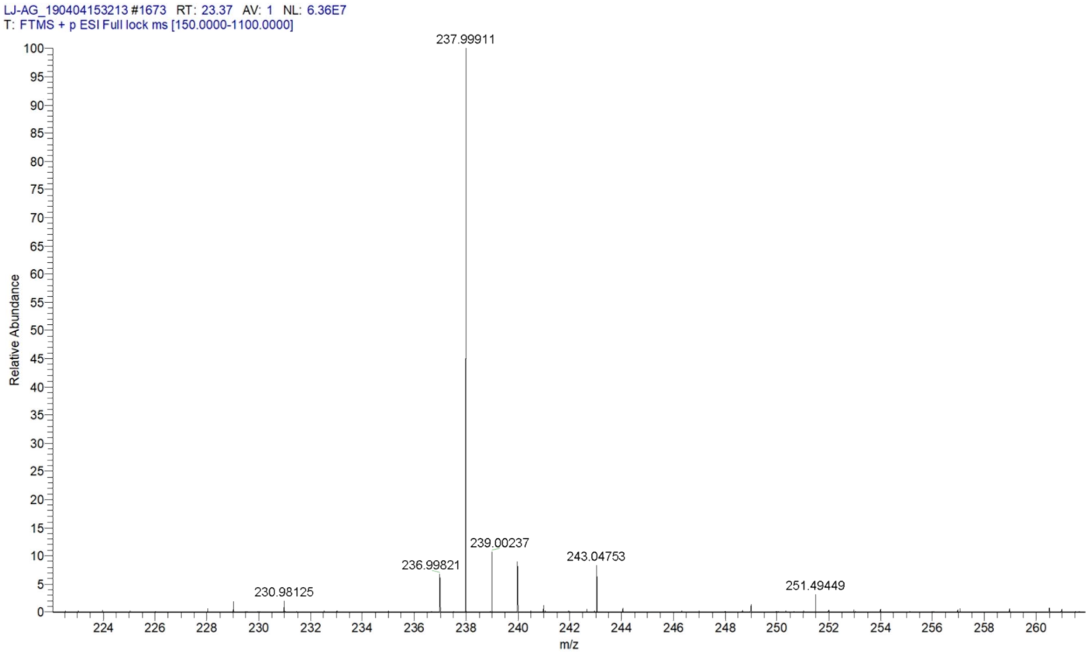

Fig. S.16. ESI-HRMS mass spectrum of 2,3-dihydrothiazolo[3,2-a]thiopyrano[3,4-d] pyridine - 5,10-dione (A-G).

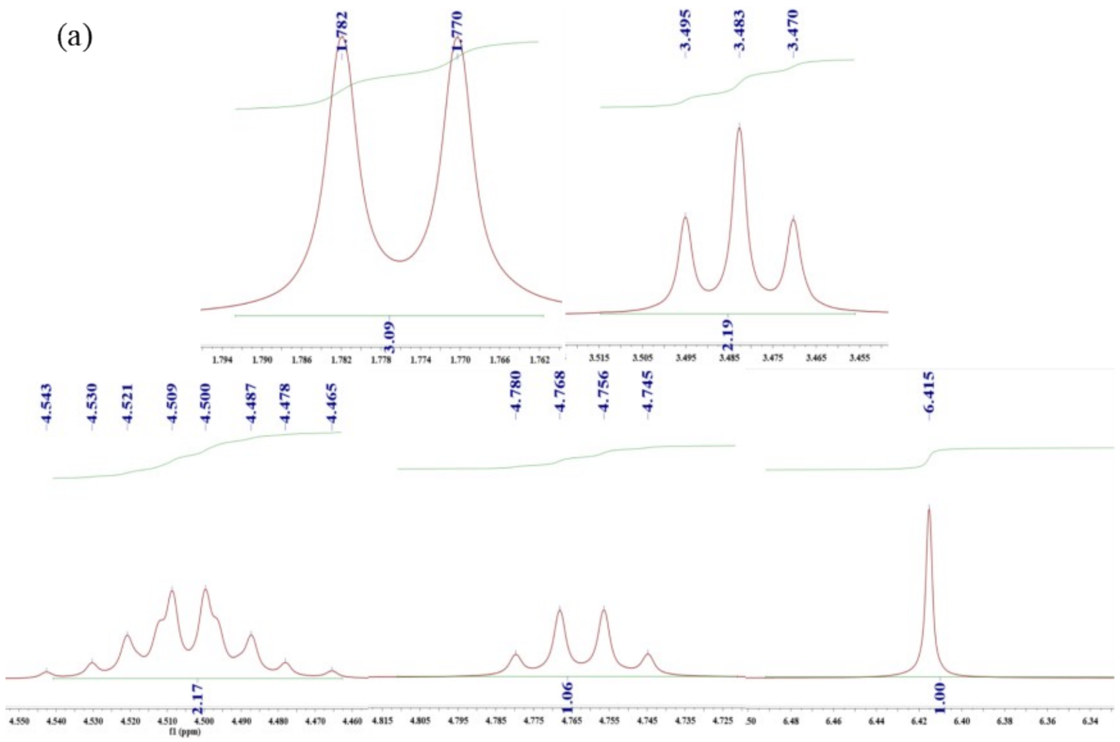

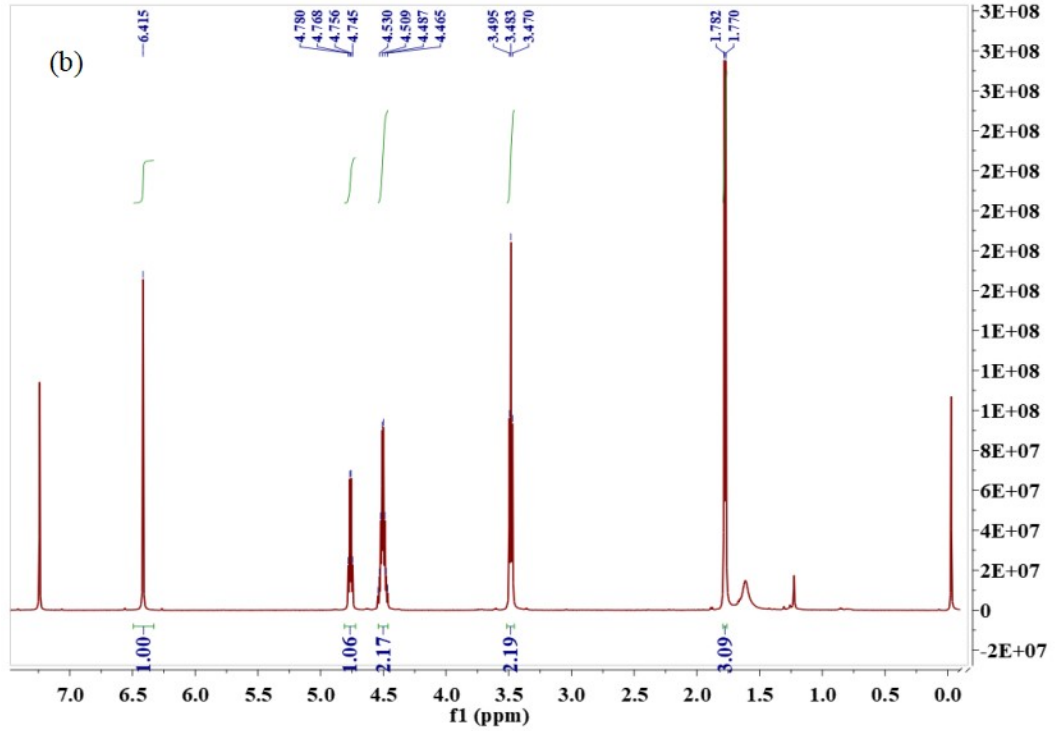

Fig. S.17. (a) is the amplified spectrum of each peak at the ^{1}H-NMR spectrum. (b) is ^{1}H-NMR spectrum of 7-methyl-2,3-dihydrothiazolo[3,2-a]-7*H*-thieno[3,4-d]pyridine-5,9-dione (A-BG) dissolved in CD_3CL.

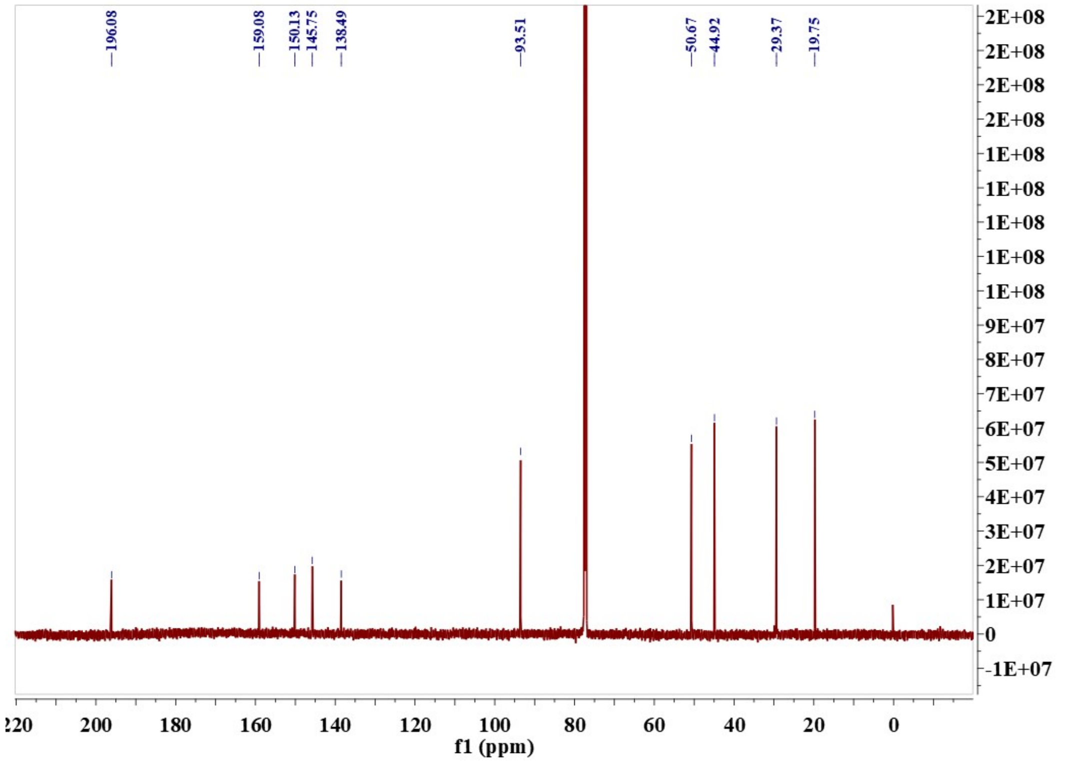

Fig. S.18.^{13}C-NMR spectrum of 7-methyl-2,3-dihydrothiazolo[3,2-a]-7*H*-thieno[3,4-d]pyridine -5,9-dione (A-BG) dissolved in CD_3CL.

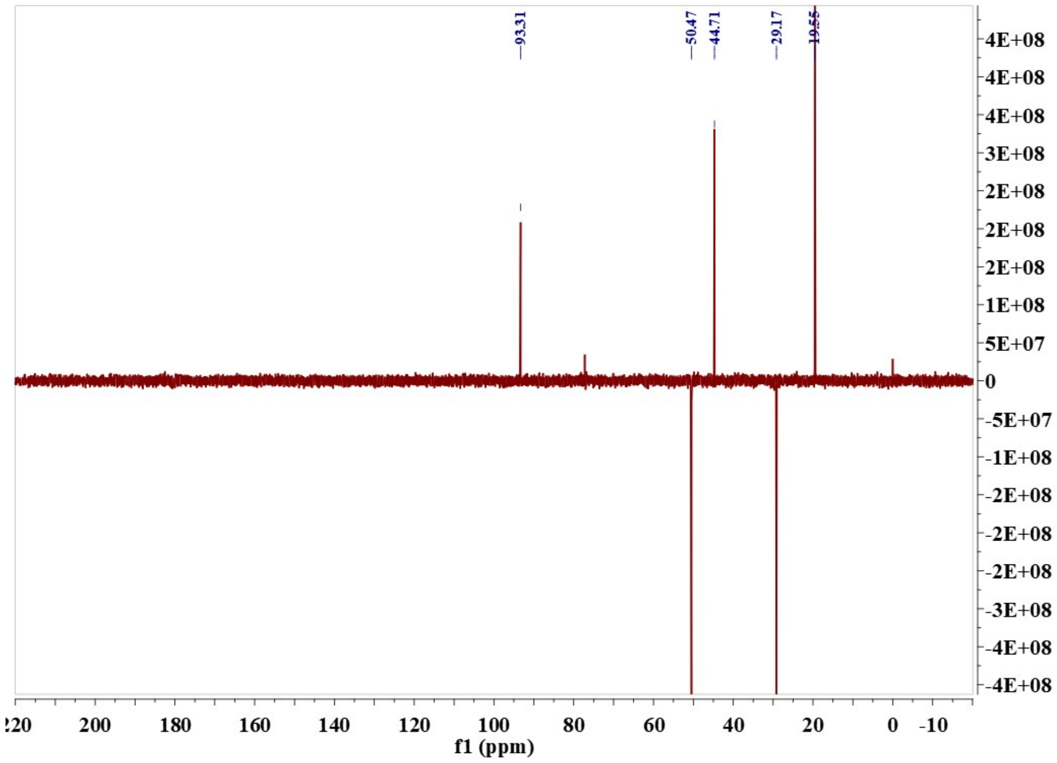

Fig. S.19. Dept 135-NMR spectrum of 7-methyl-2,3-dihydrothiazolo[3,2-a]-7*H*-thieno[3,4-d] pyridine -5,9-dione (A-BG) dissolved in CD_3CL.

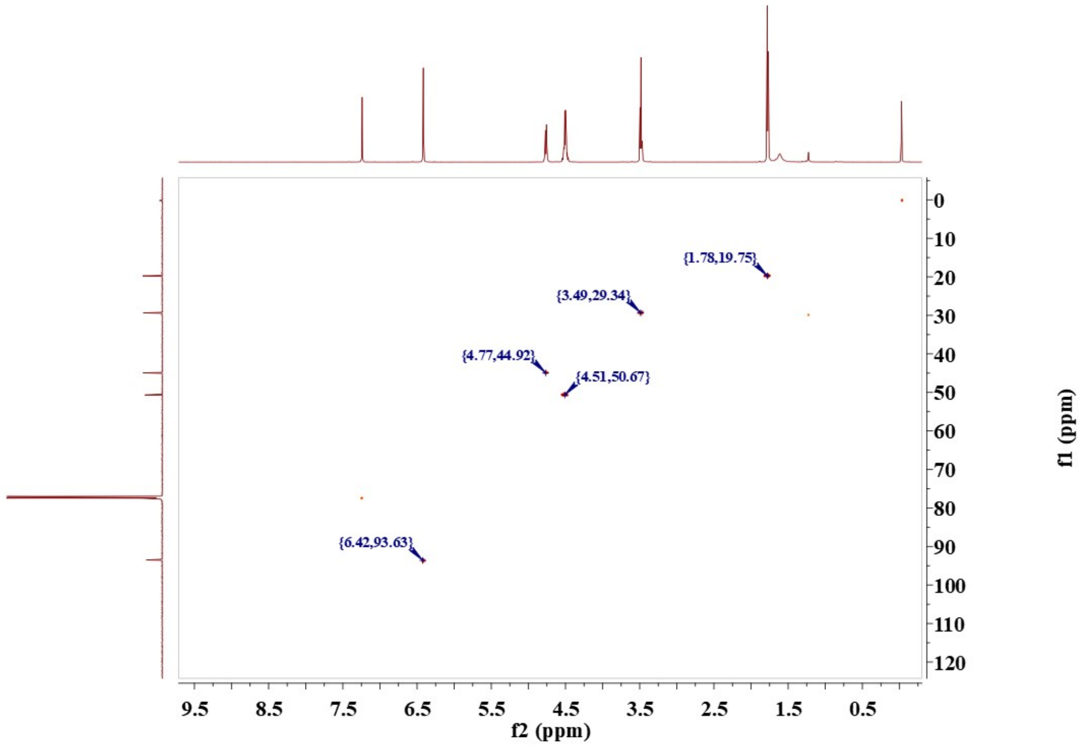

Fig. S.20. HSQC-NMR spectrum of 7-methyl-2,3-dihydrothiazolo[3,2-a]-7*H*-thieno[3,4-d] pyridine-5,9-dione (A-BG) dissolved in CD_3CL.

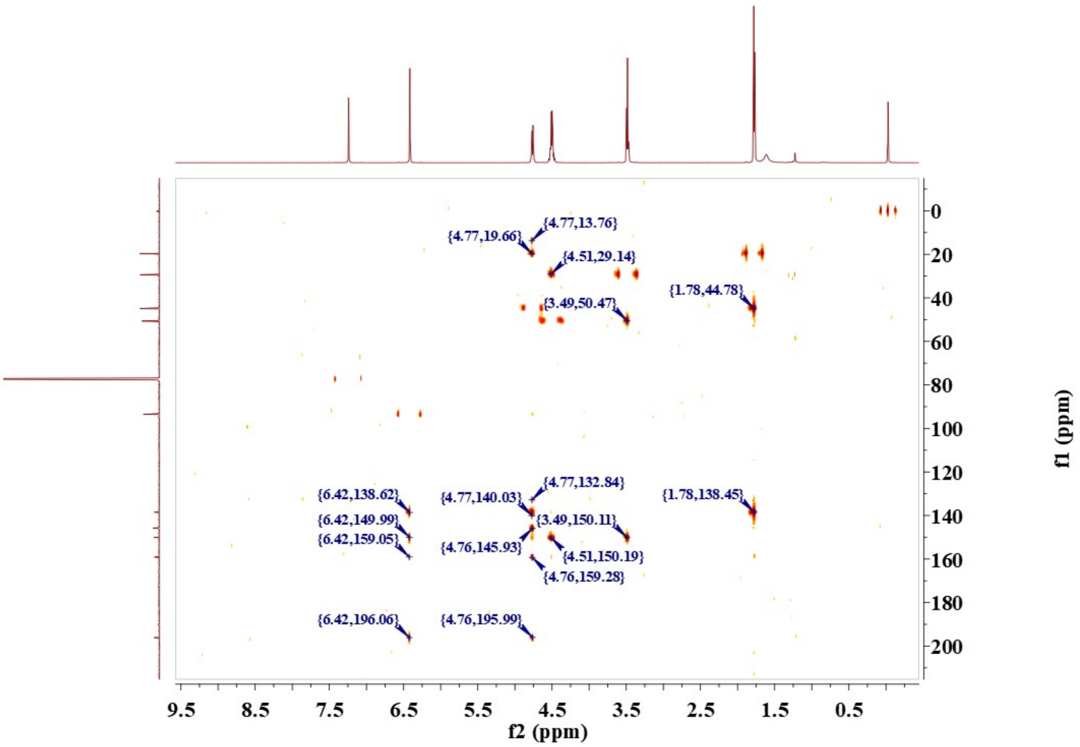

Fig. S.21. HMBC-NMR spectrum of 7-methyl-2,3-dihydrothiazolo[3,2-a]-7*H*-thieno[3,4-d] pyridine-5,9-dione (A-BG) dissolved in CD_3CL.

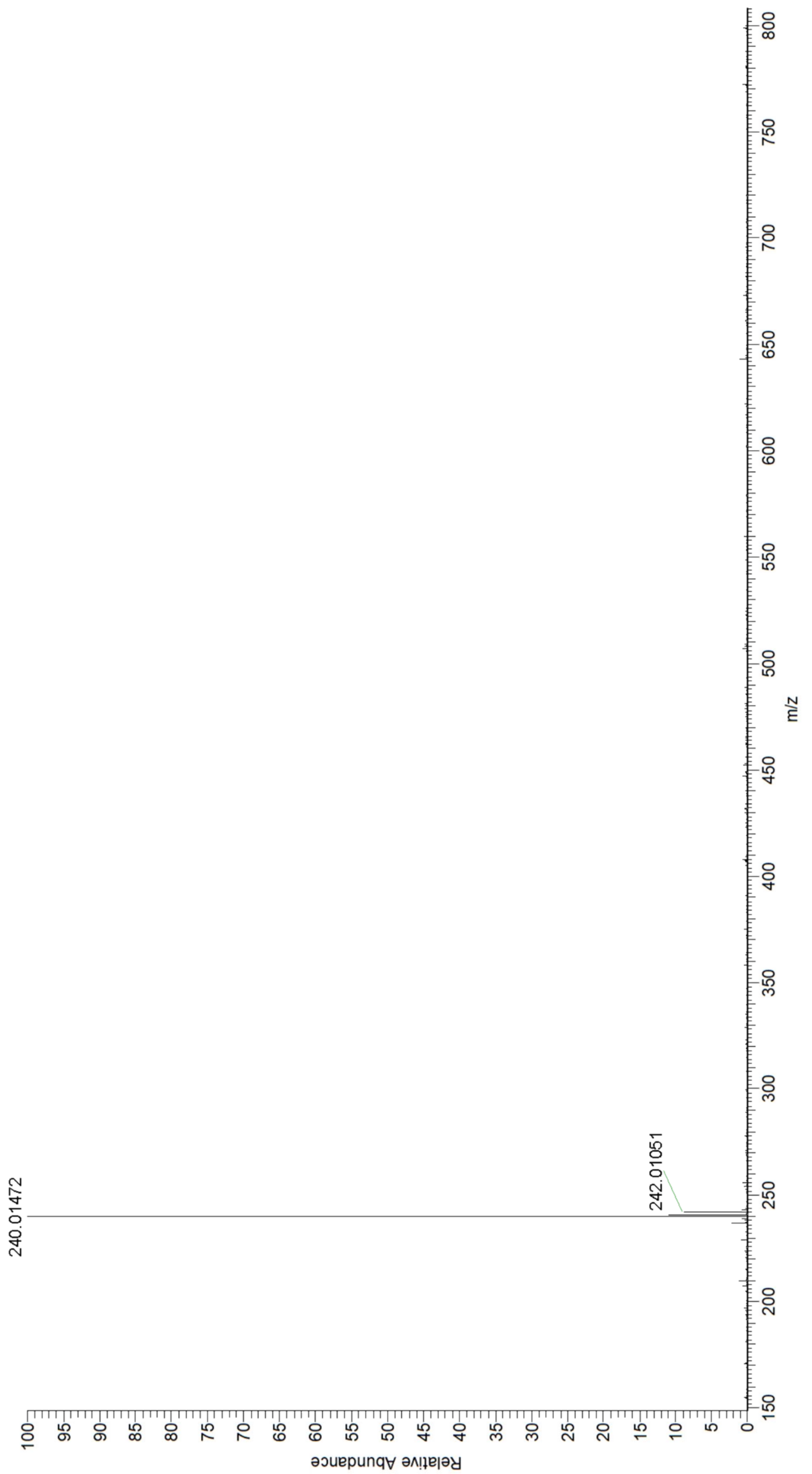

Fig. S.22. ESI-HRMS mass spectrum of 7-methyl-2,3-dihydrothiazolo[3,2-a]-7*H*-thieno[3,4-d] pyridine-5,9-dione (A-BG).

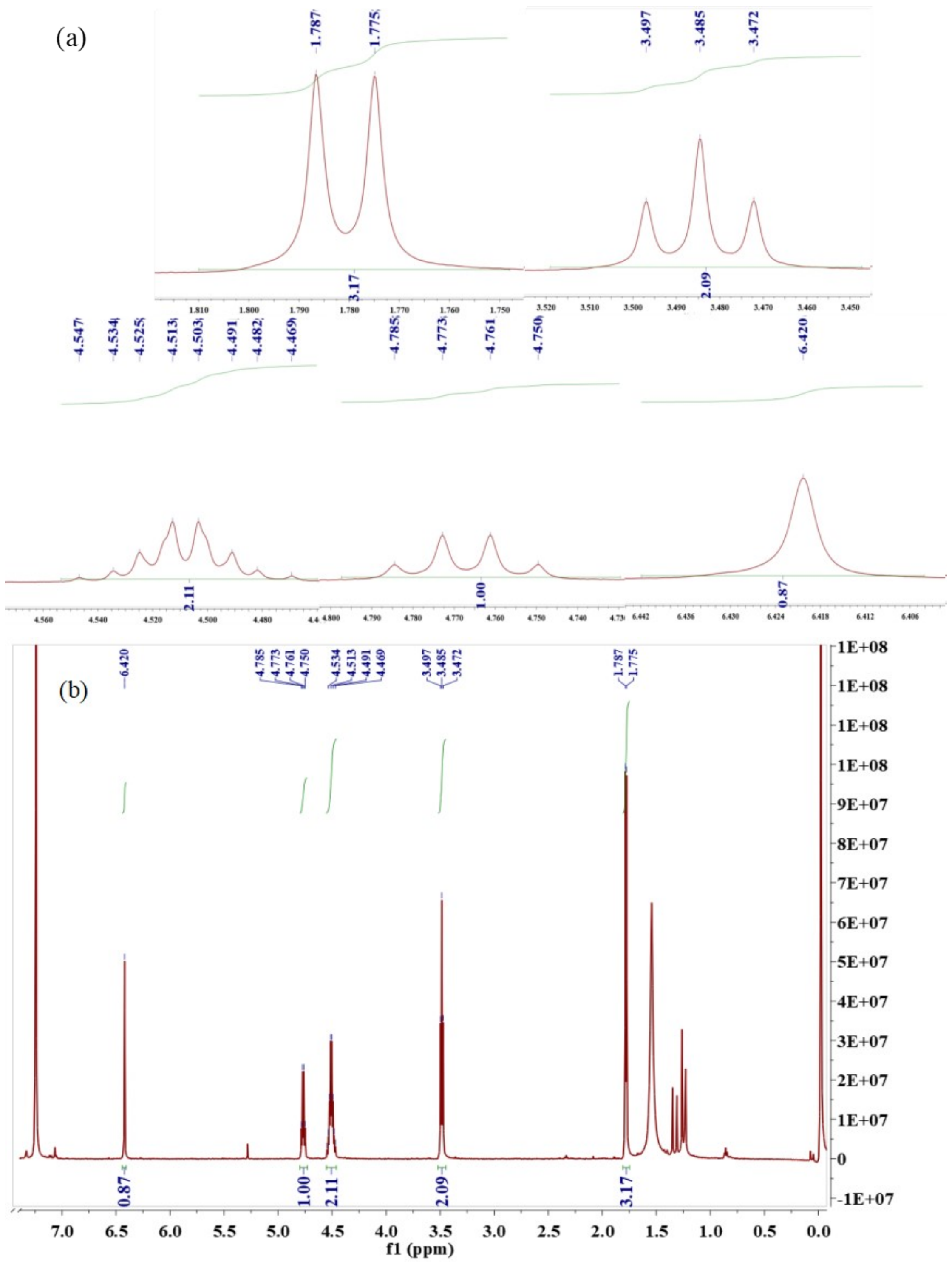

Fig. S.23. (a) is the amplified spectrum of each peak at the ^{1}H-NMR spectrum. (b) is ^{1}H-NMR spectrum of 7-methyl-2,3-dihydrothiazolo[3,2-a]-7*H*-thieno[3,4-d]pyridine-5,9-dione (B-BG-1) dissolved in CD_3CL.

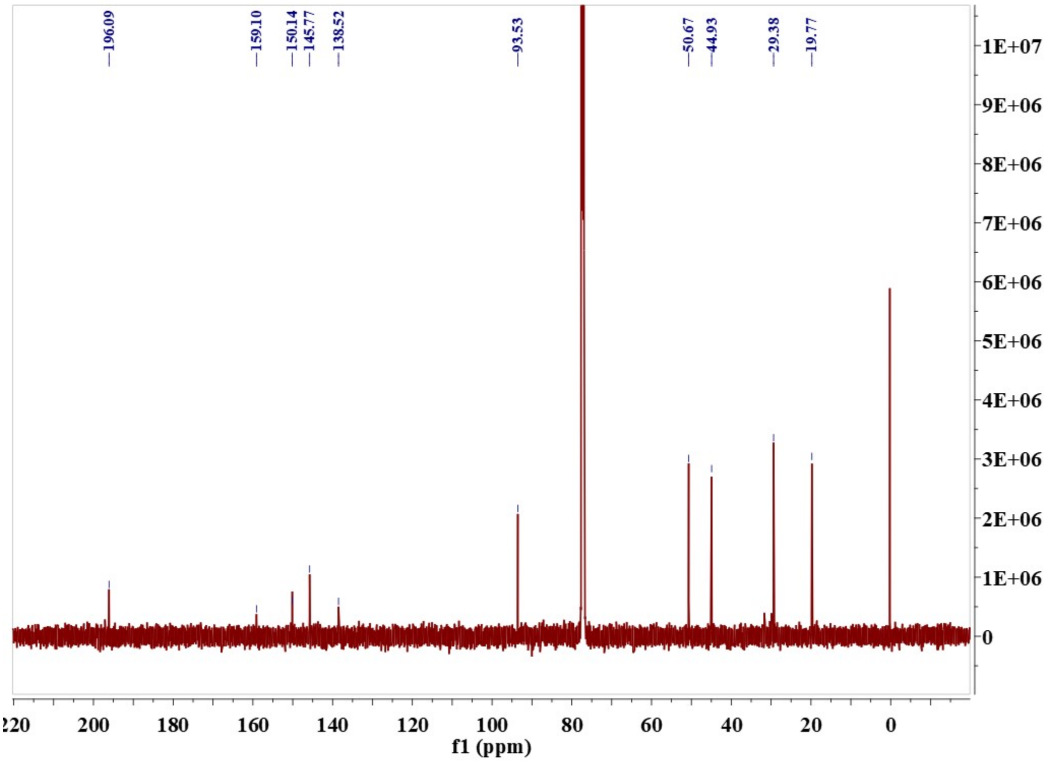

Fig. S.24. ^{13}C-NMR spectrum of 7-methyl-2,3-dihydrothiazolo[3,2-a]-7*H*-thieno[3,4-d] Pyridine-5,9-dione (B-BG-1) dissolved in CD_3CL.

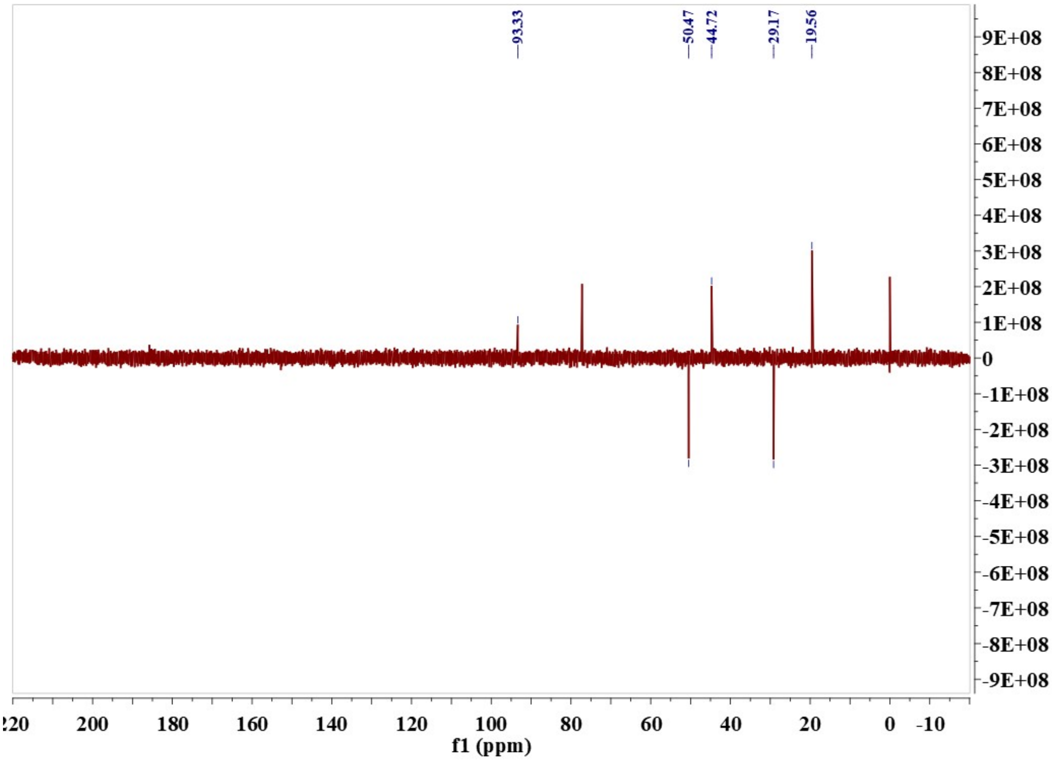

Fig. S.25. Dept 135-NMR spectrum of 7-methyl-2,3-dihydrothiazolo[3,2-a]-7*H*-thieno[3,4-d] pyridine -5,9-dione (B-BG-1) dissolved in CD_3CL.

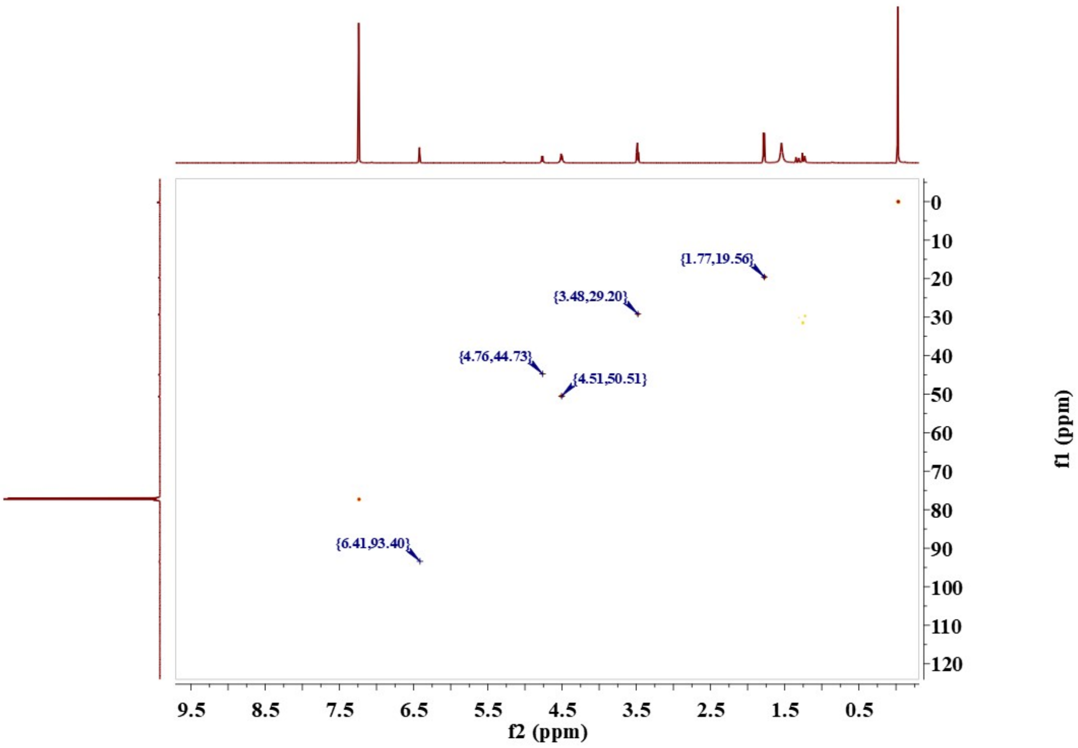

Fig. S.26. HSQC-NMR spectrum of 7-methyl-2,3-dihydrothiazolo[3,2-a]-7*H*-thieno[3,4-d] pyridine-5,9-dione (B-BG-1) dissolved in CD_3CL.

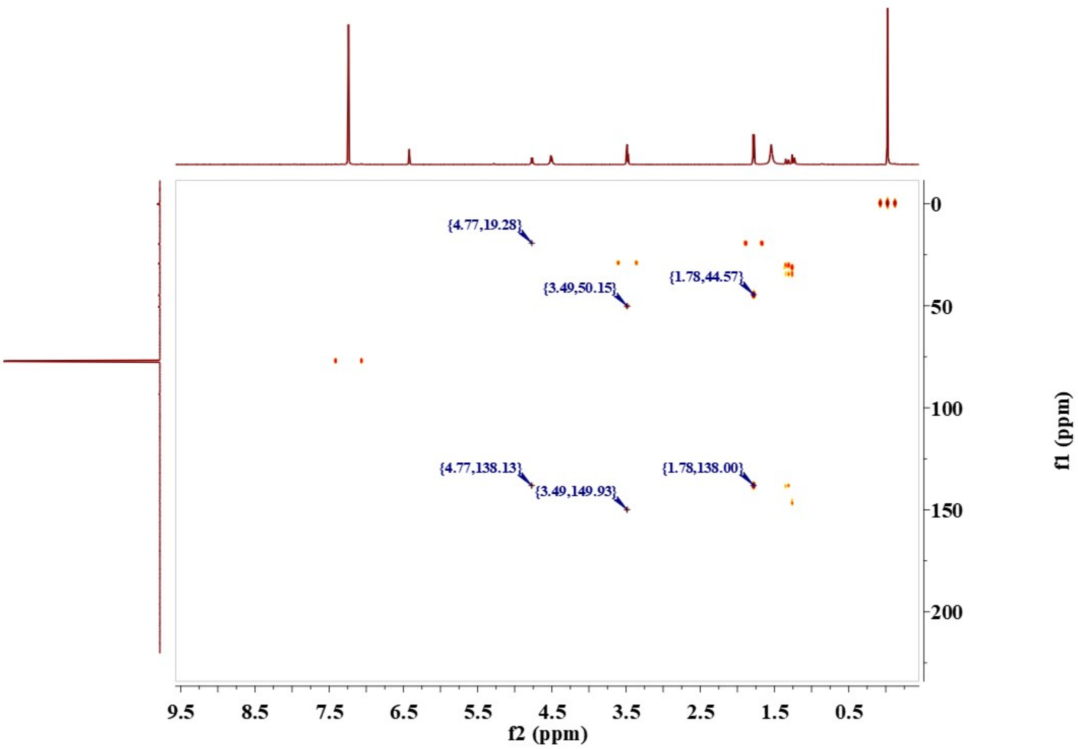

Fig. S.27. HMBC-NMR spectrum of7-methyl-2,3-dihydrothiazolo[3,2-a]-7*H*-thieno[3,4-d] pyridine-5,9-dione (B-BG-1) dissolved in CD_3CL.

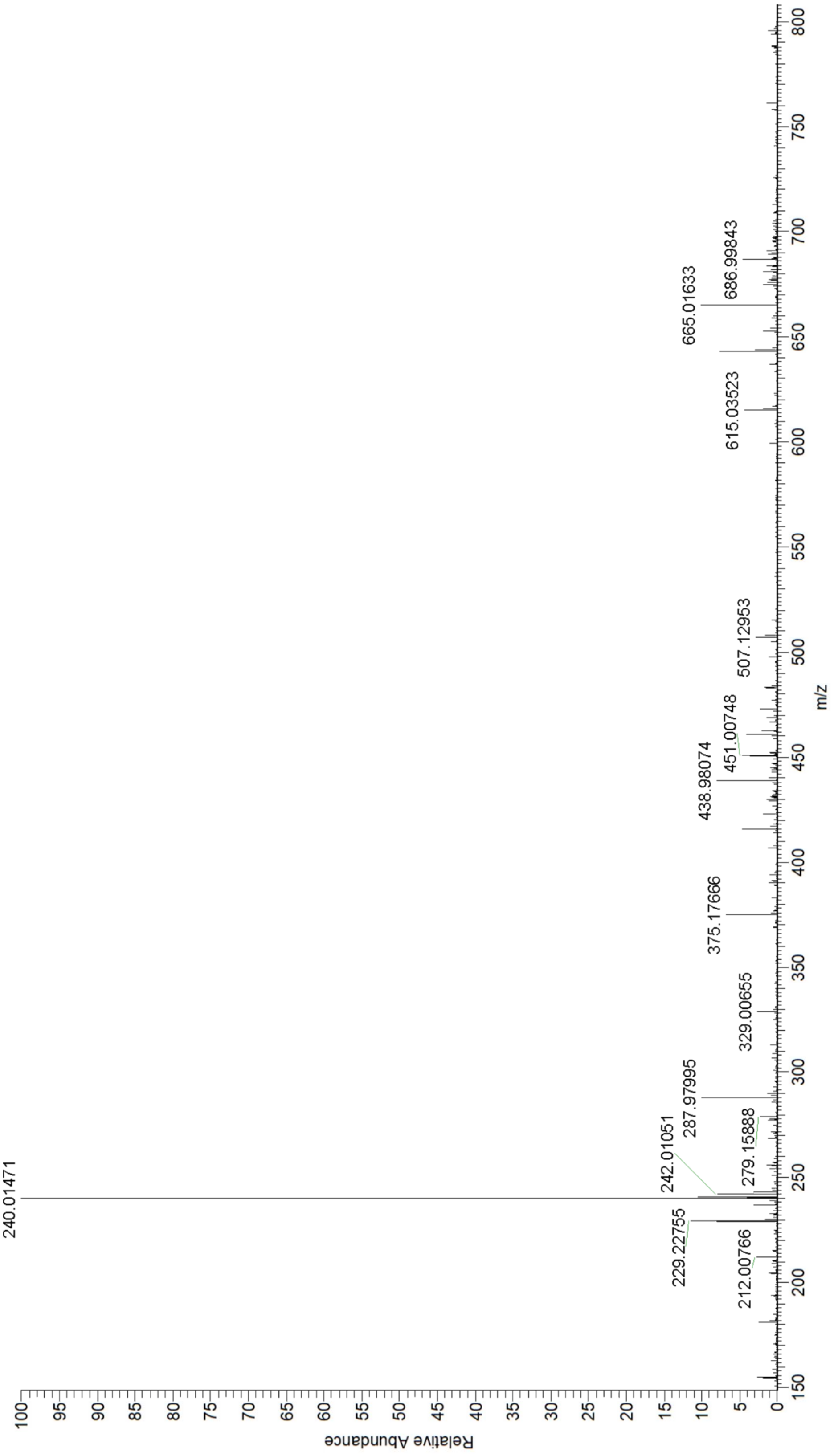

Fig. S.28. ESI-HRMS mass spectrum of 7-methyl-2,3-dihydrothiazolo[3,2-a]-7*H*-thieno[3,4-d] pyridine-5,9-dione (B-BG-1) dissolved.

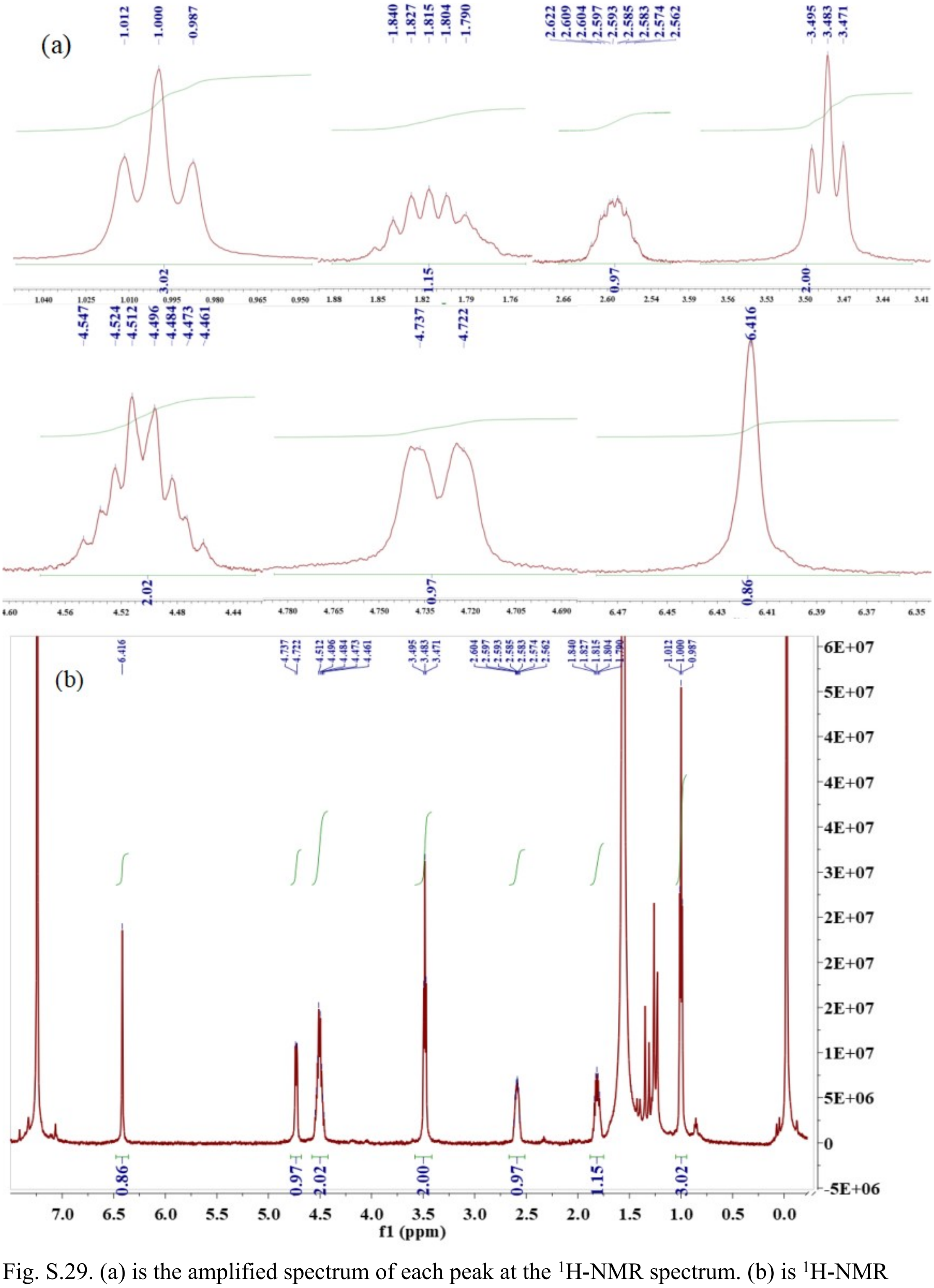

Fig. S.29. (a) is the amplified spectrum of each peak at the ^{1}H-NMR spectrum. (b) is ^{1}H-NMR spectrum of 7-ethyl-2,3-dihydrothiazolo[3,2-a]-7*H*-thieno[3,4-d] pyridine-5,9-dione (B-G-2) dissolved in CD_3CL.

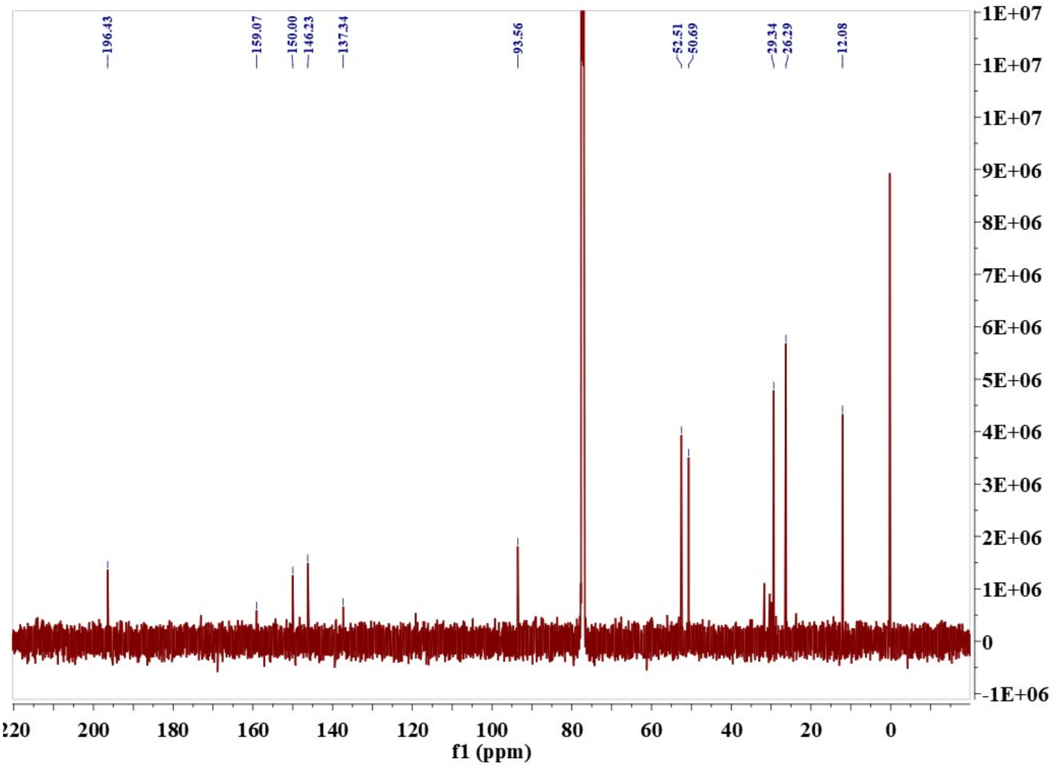

Fig. S.30. ^{13}C-NMR spectrum of 7-ethyl-2,3-dihydrothiazolo[3,2-a]-7*H*-thieno[3,4-d] pyridine-5,9-dione (B-G-2) dissolved in CD_3CL.

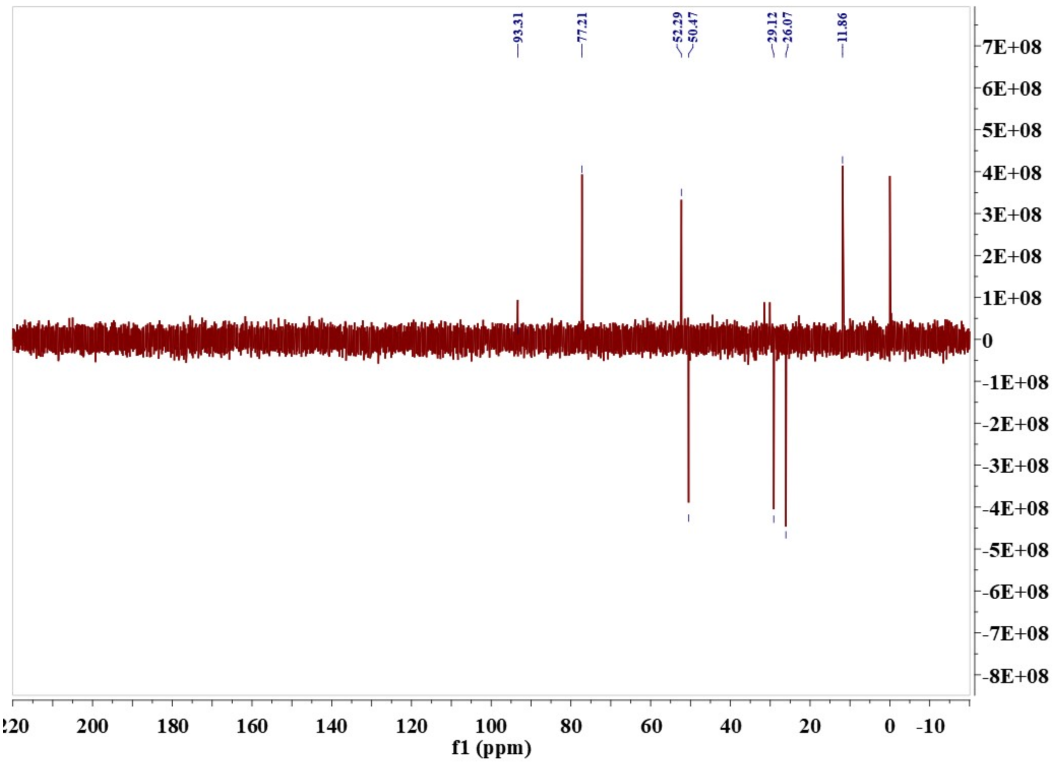

Fig. S.31. Dept 135-NMR spectrum of 7-ethyl-2,3-dihydrothiazolo[3,2-a]-7*H*-thieno[3,4-d] pyridine-5,9-dione (B-G-2) dissolved in CD_3CL.

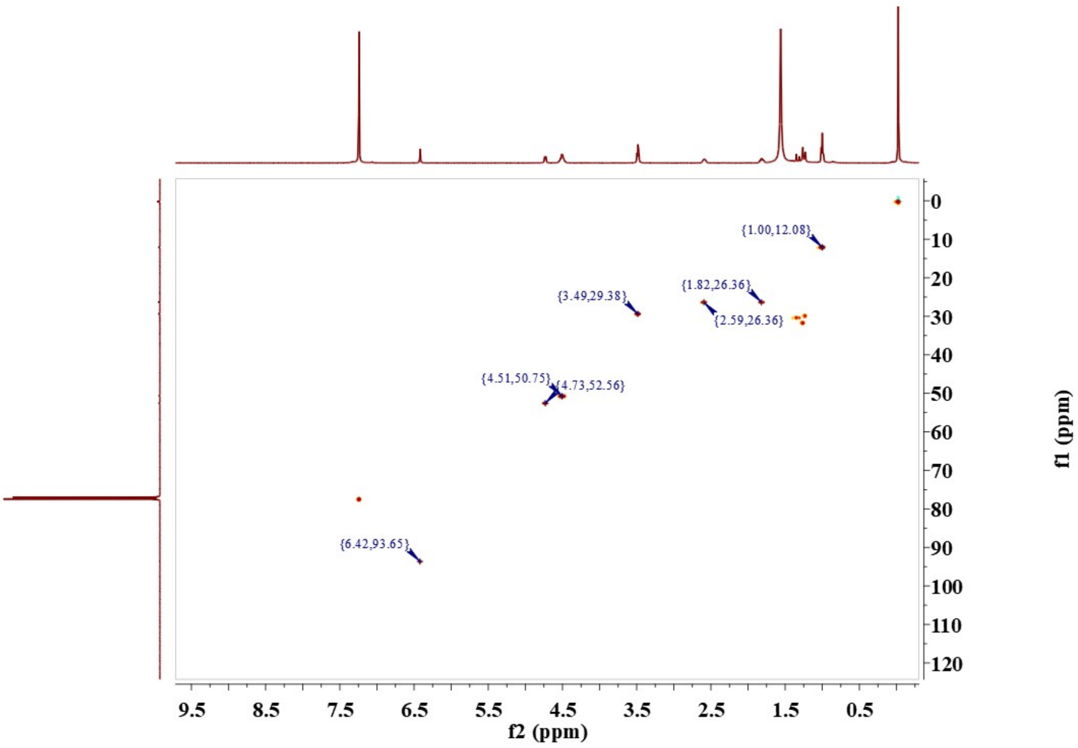

Fig. S.32. HSQC-NMR spectrum of 7-ethyl-2,3-dihydrothiazolo[3,2-a]-7*H*-thieno[3,4-d] pyridine-5,9-dione (B-G-2) dissolved in CD_3CL.

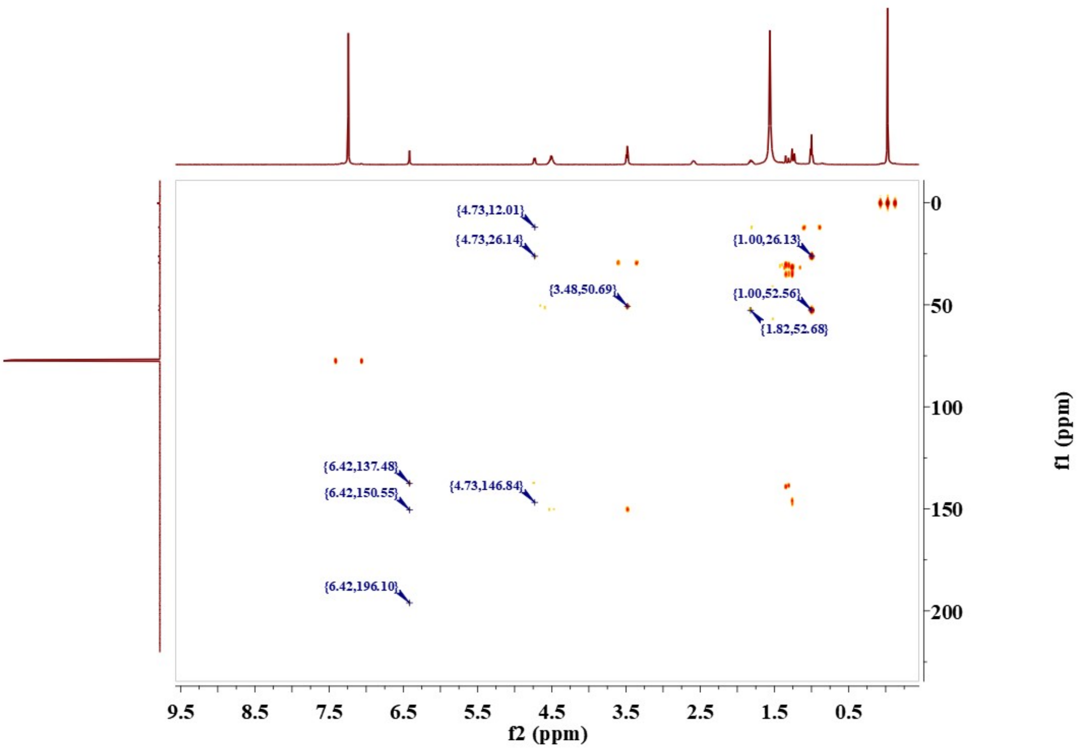

Fig. S.33. HMBC-NMR spectrum of 7-ethyl-2,3-dihydrothiazolo[3,2-a]-7*H*-thieno[3,4-d] pyridine-5,9-dione (B-G-2) dissolved in CD_3CL.

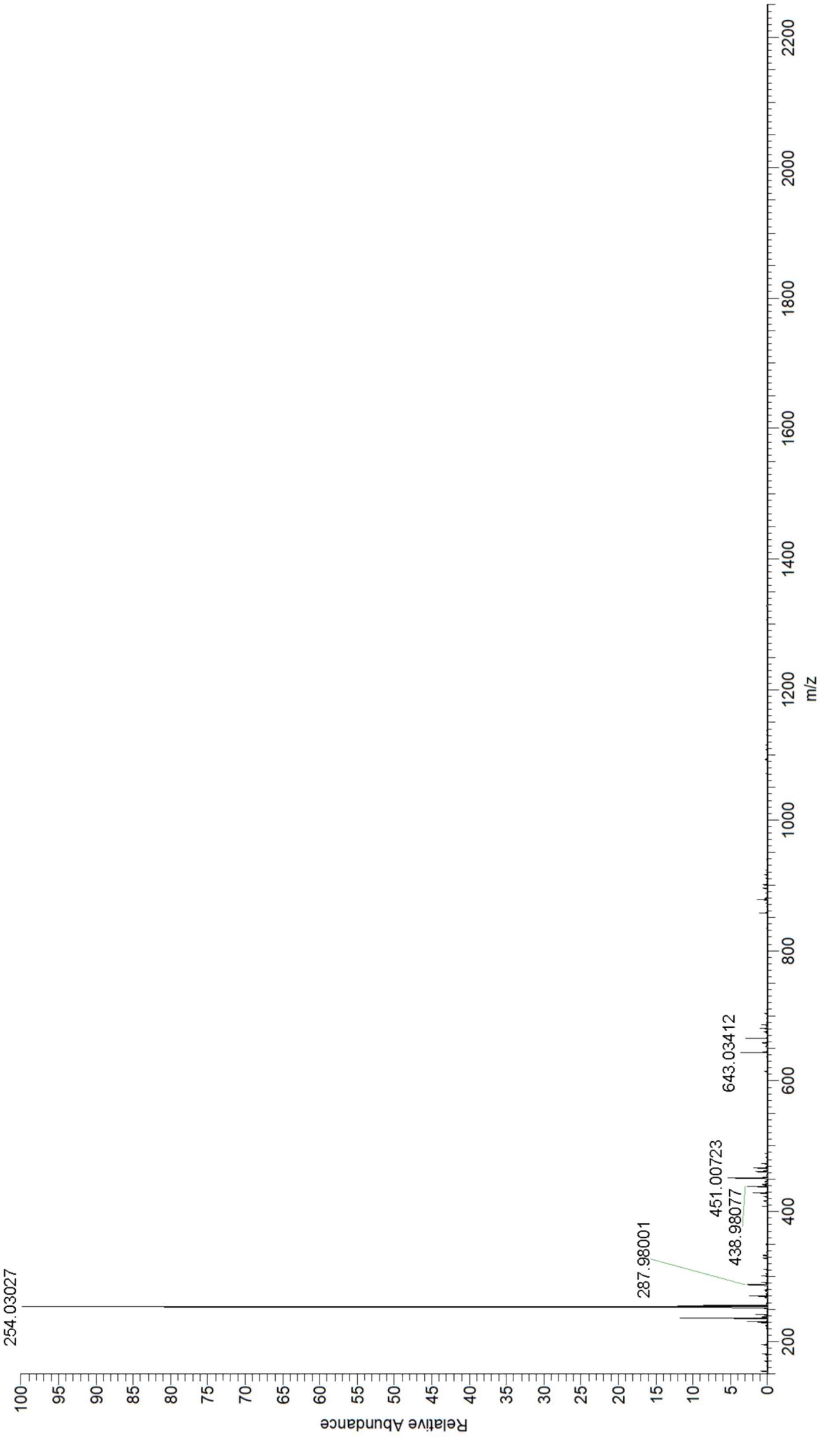

Fig. S.34. ESI-HRMS mass spectrum of 7-ethyl-2,3-dihydrothiazolo[3,2-a]-7*H*-thieno[3,4-d] pyridine-5,9-dione (B-G-2).

Table 2. The differences in the Substituents on amide α-C and fluorescence properties including $\lambda_{ex/em}$ and QY (%) of CDs-NAC, CDs-GSH, and all fluorophores separated from CDs-NAC and CDs-GSH.

Sample	Substituents on amide α-C	λ_{ex}	λ_{em}	QY (%)
CDs-NAC	*	365	440	29.83%
CDs-GSH	*	365	440	26.23%
A-B	H	367	441	55.36%
A-G	-S-CH=CH-	414	489	44.30%
A-BG	-S-CH(CH_3)-	370	468	36.63%
B-BG-1	-S-CH(CH_3)-	371	468	35.11%
B-BG-2	-S-CHCH$_2$(CH_3)-	371	468	44.57%

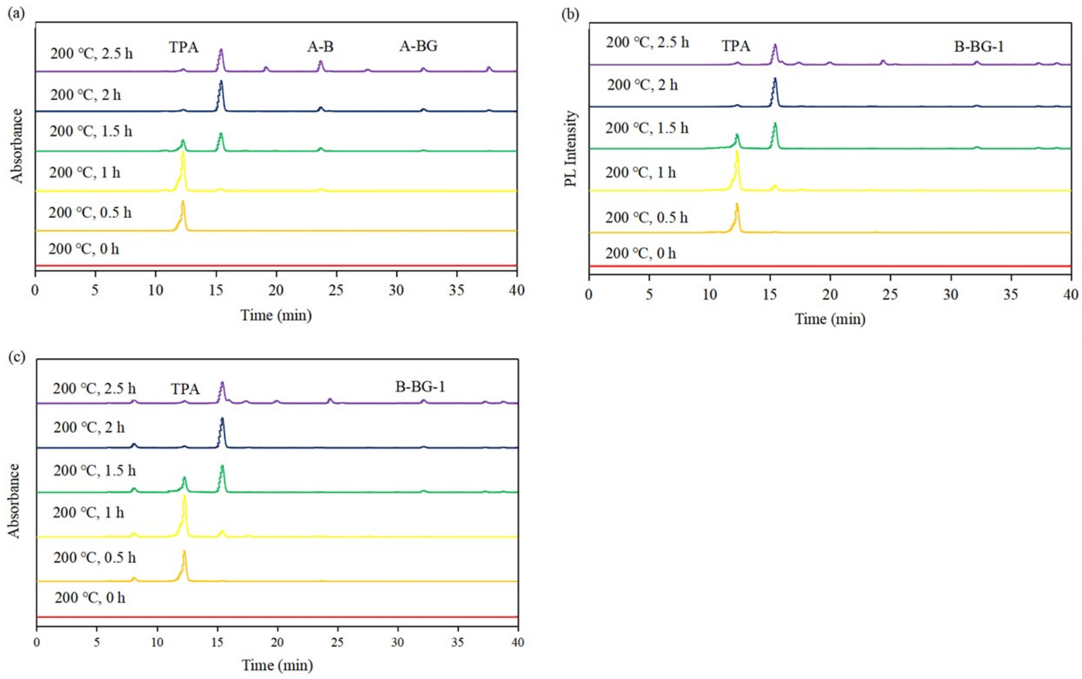

Fig. S.35. (a) The absorption chromatograms of CDs-NAC at the different reaction time of the second step monitored at 365 nm. (b) the fluorescence chromatograms monitored at 365 nm/440 nm and (c) its corresponding absorption chromatograms of CDs-GSH at the different reaction time of the second step monitored at 365 nm. All of these were carried out under the same HPLC conditions. Column temperature: 25 ℃; Eluent ratio and time: acetonitrile and water were used as two phases and all samples were eluted with acetonitrile whose concentration is from 15% to 55% during 45 min.

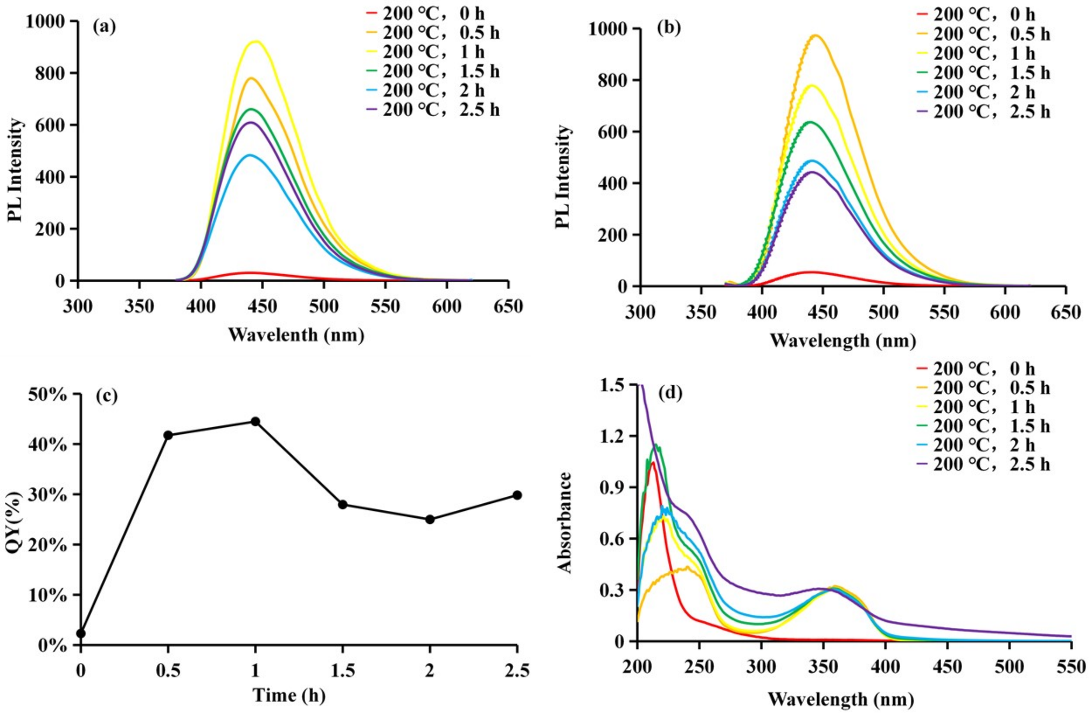

Fig. S.36. (a) Fluorescence spectra at optimal excitation wavelengths of CDs-NAC prepared at 200 °C for various reaction times (0, 0.5, 1, 1.5, 2 and 2.5 h). (b) Fluorescence spectra at optimal excitation wavelengths, (c) QY and (d) UV–visible absorption spectra of CDs-GSH prepared at 200 °C for various reaction times (0, 0.5, 1, 1.5, 2 and 2.5 h).

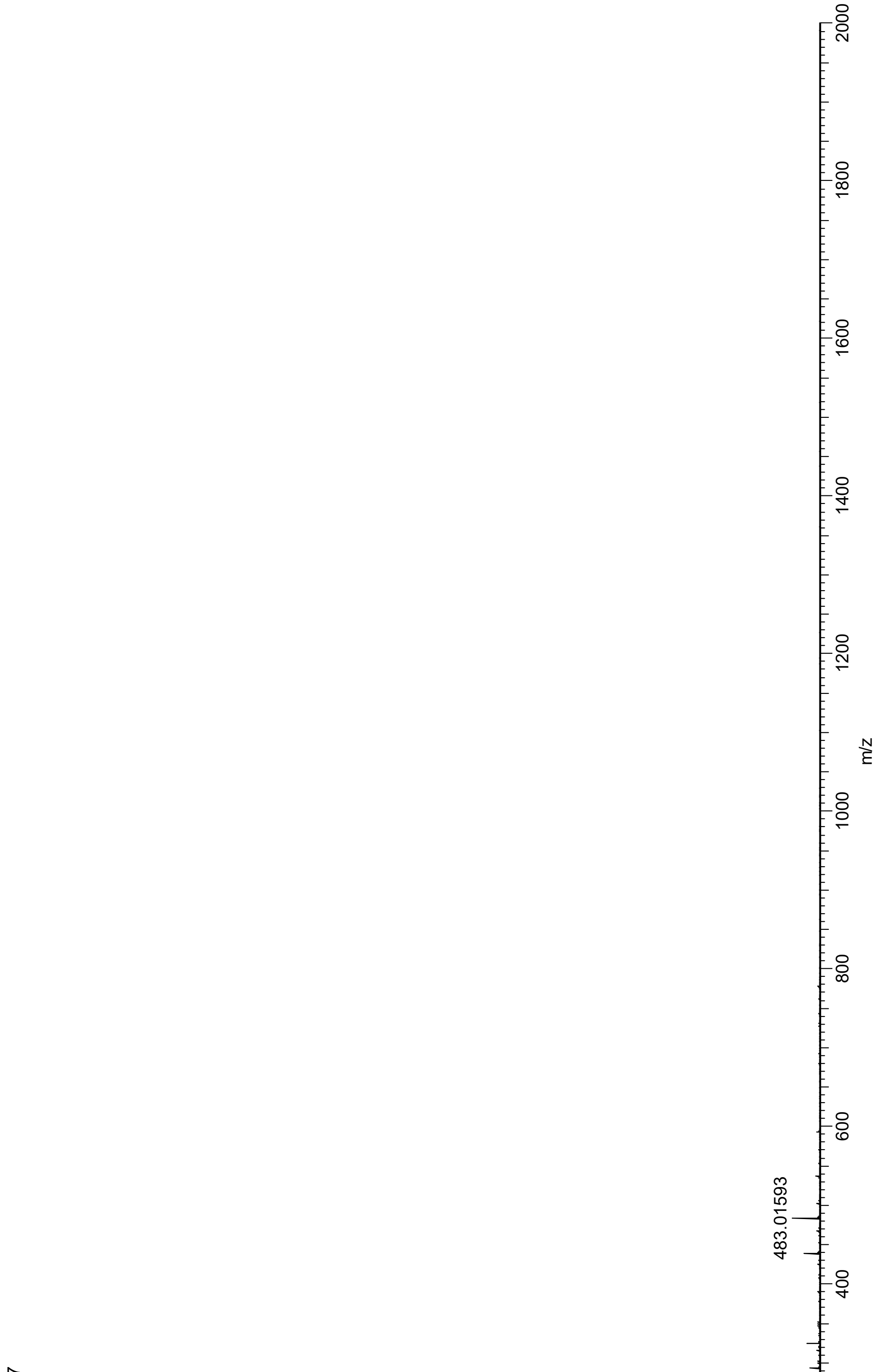

Fig. S.37. ESI-HRMS mass spectrum of TPA from NCA-CDs. The peek of 242.01147 is $[TPA+H]^+$ and the peek of 483.01593 is $[2TPA+H]^+$.

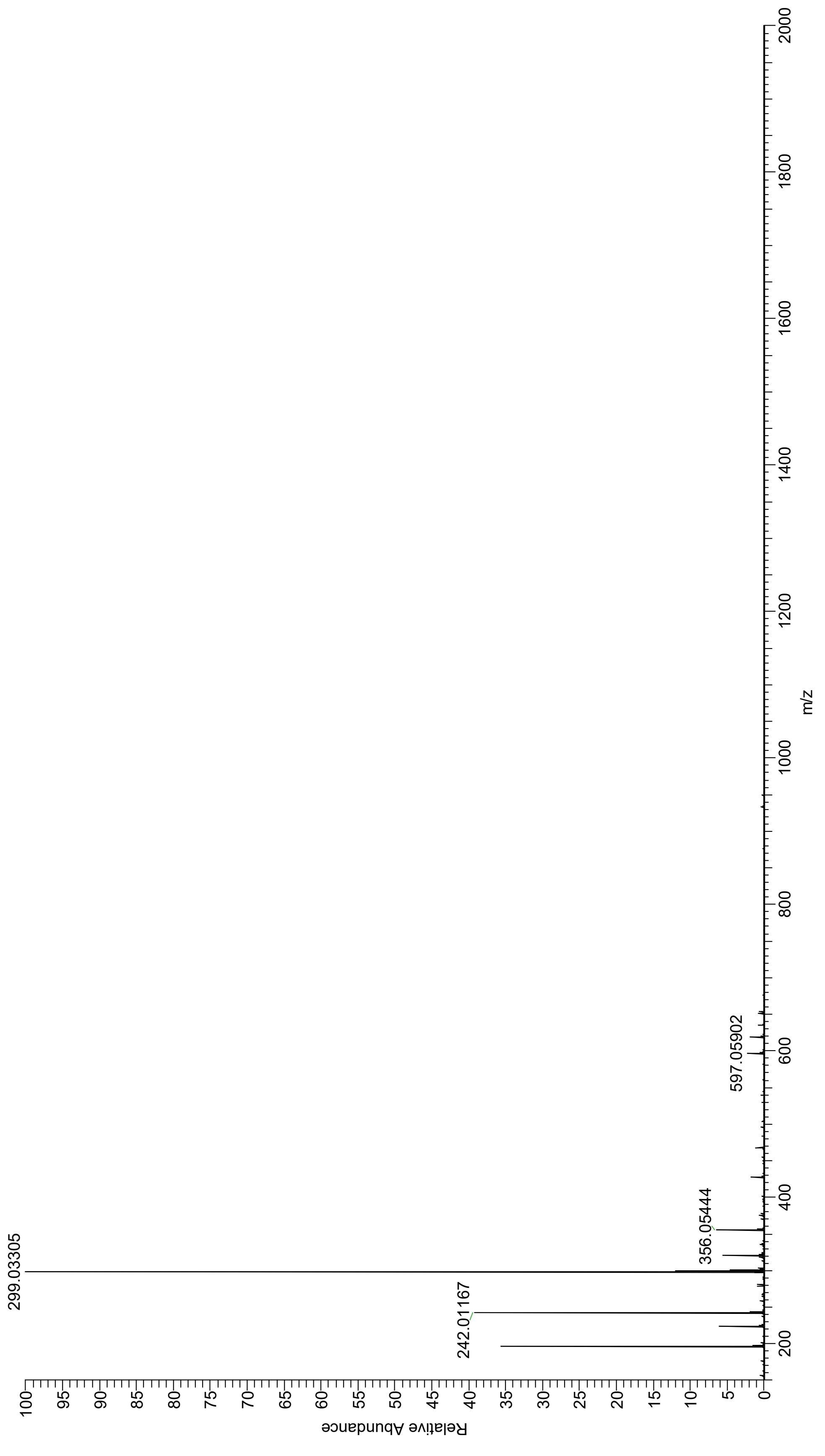

Fig. S.38. ESI-HRMS mass spectrum of TPA from CDs-GSH. The peek of 242.01167 is [TPA+H]$^{+}$.

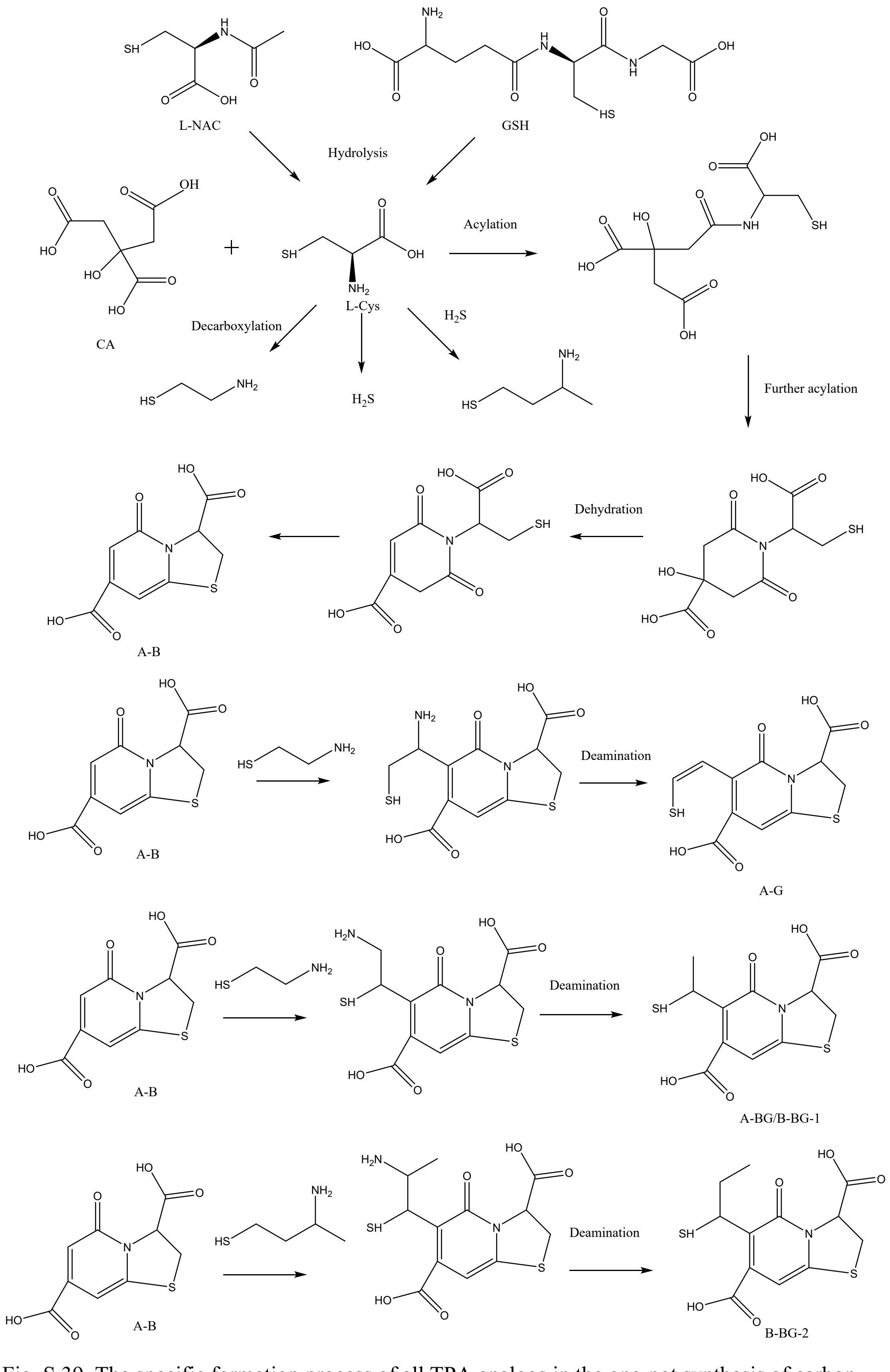

Fig. S.39. The specific formation process of all TPA analogs in the one-pot synthesis of carbon dots and all reactions were at a temperature of 200 ℃.

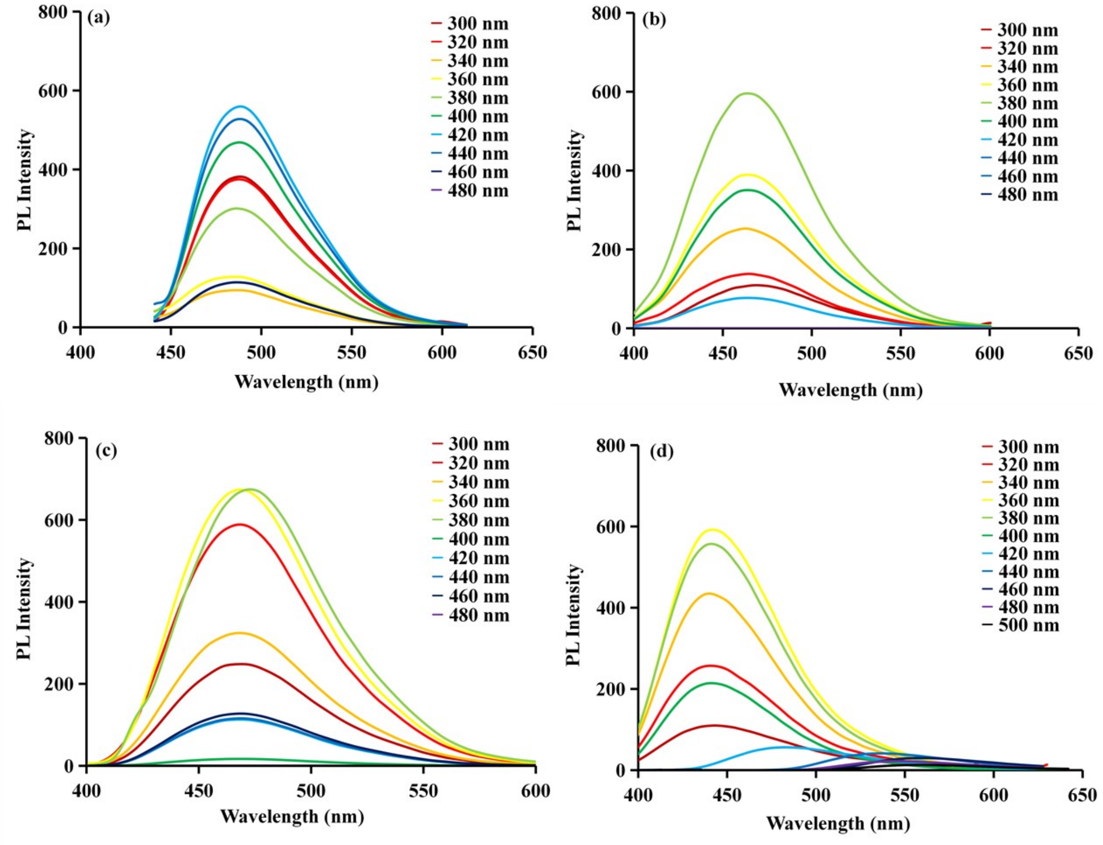

Fig. S.40. Fluorescence spectra of (a) A-G, (b) B-BG-1, (c) B-BG-2 and (d) CDs-GSH.

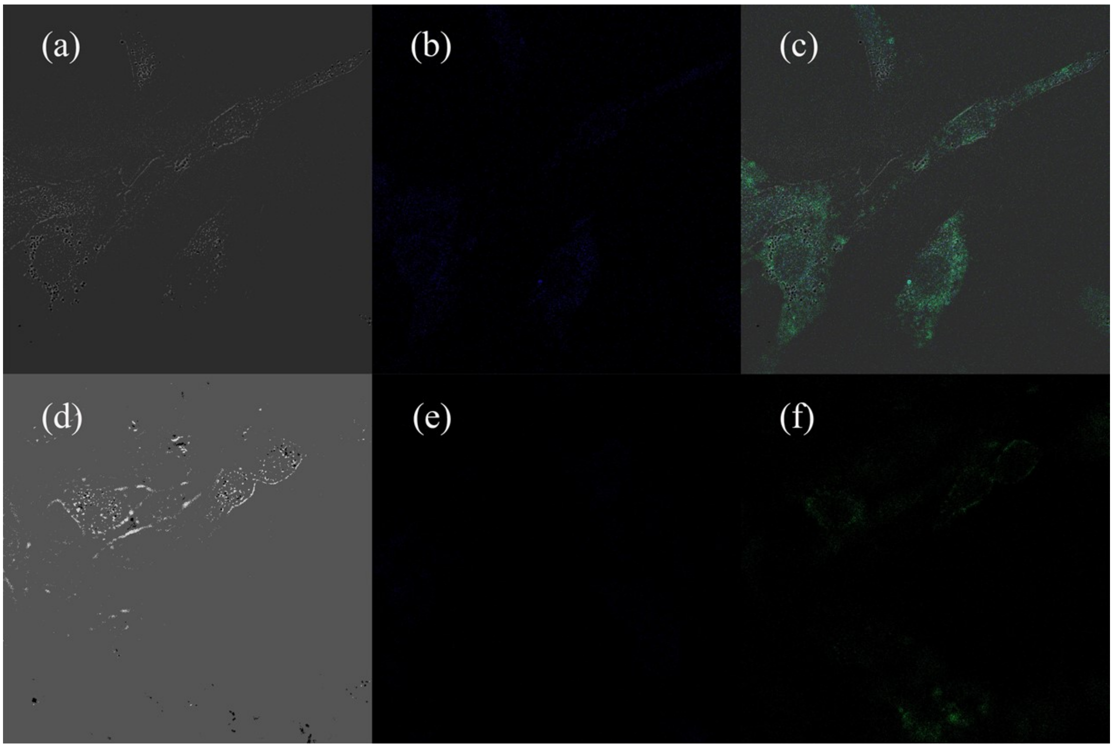

Fig. S.41. Cell imaging under laser microscope after incubation of CDs-NAC and CDs-GSH with GES-1 cells. (a,b,c) after incubation of CDs-NAC with L02 cells, images were taken at bright

field, $\lambda_{ex/em}$=408/515nm and $\lambda_{ex/em}$=488/590nm, respectively.(d,e,f) after incubation of CDs-GSH with L02 cells, images were taken at bright field, $\lambda_{ex/em}$=408/515nm and $\lambda^{ex/em}$=488/590nm, respectively. Administration Concentration :100 μg / mL.

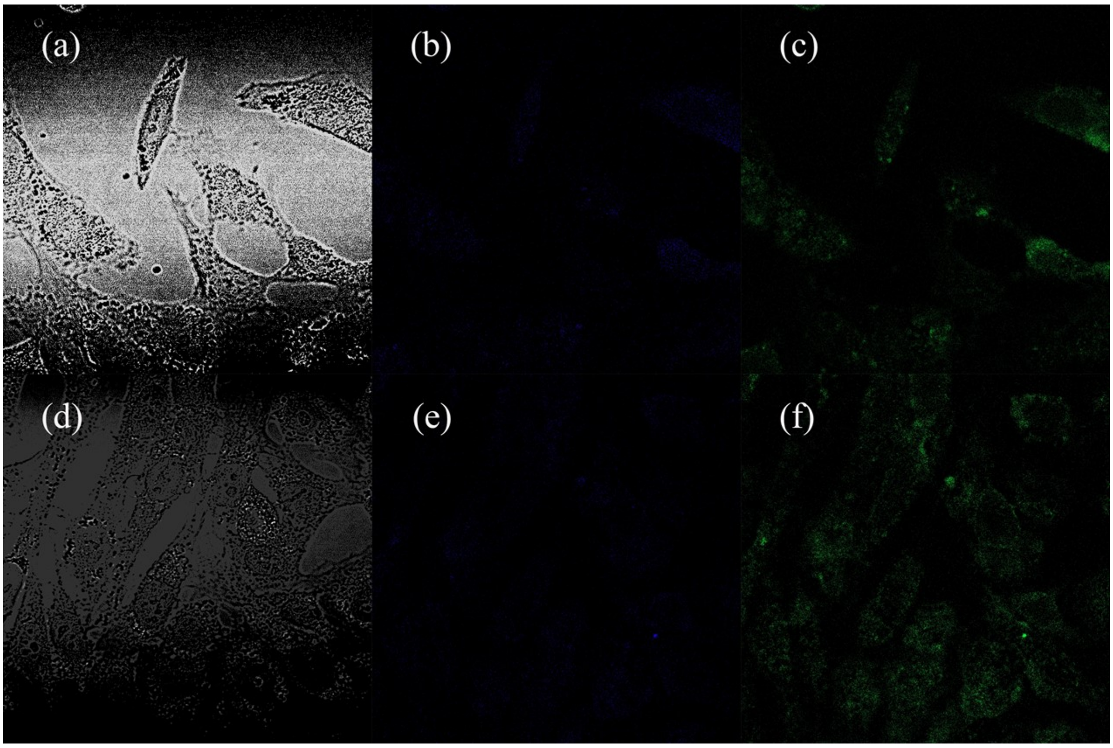

Fig. S.42. Cell imaging under laser microscope after incubation of CDs-NAC and CDs-GSH with GES-1 cells. (a,b,c) after incubation of CDs-NAC with L02 cells, images were taken at bright field, $\lambda_{ex/em}$=408/515nm and $\lambda_{ex/em}$=488/590nm, respectively.(d,e,f) after incubation of CDs-GSH with L02 cells, images were taken at bright field, $\lambda_{ex/em}$=408/515nm and $\lambda_{ex/em}$=488/590nm, respectively. Administration Concentration :200 μg / mL.

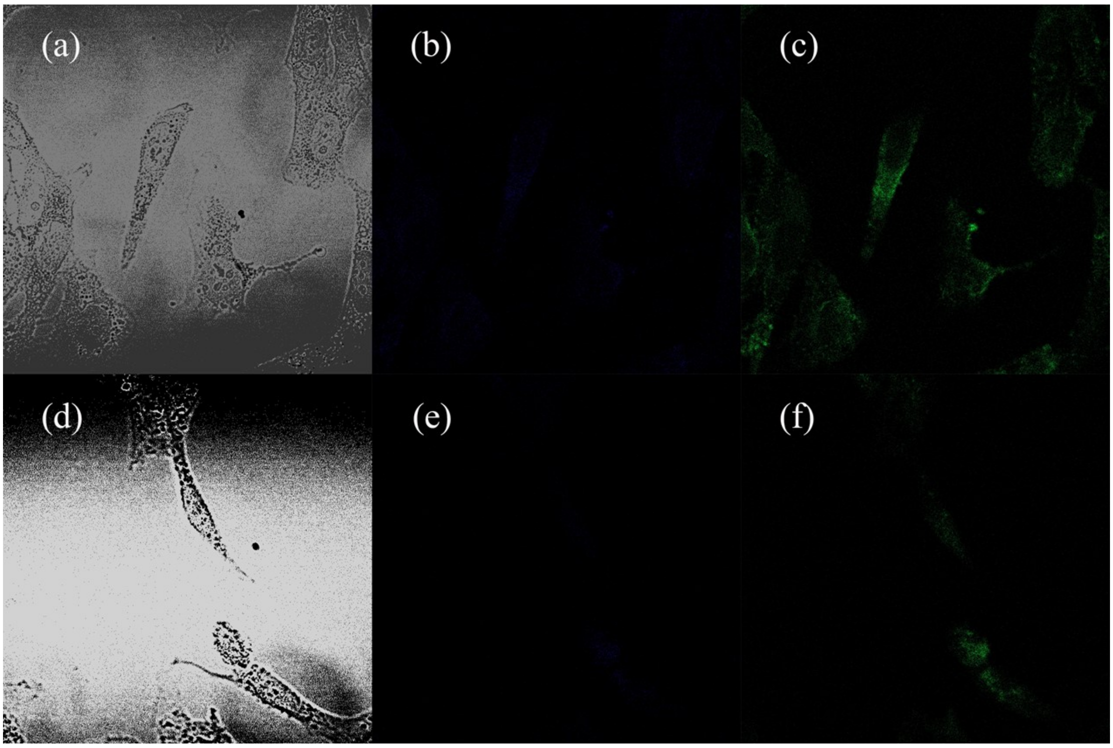

Fig. S.43. Cell imaging under laser microscope after incubation of CDs-NAC and CDs-GSH with GES-1 cells. (a,b,c) after incubation of CDs-NAC with L02 cells, images were taken at bright field, λex/em=$_{408/515}$nm and $\lambda_{ex/em}$=488/590nm, respectively.(d,e,f) after incubation of CDs-GSH with L02 cells, images were taken at bright field, $\lambda_{ex/em}$=408/515nm and $\lambda_{ex/em}$=488/590nm, respectively. Administration Concentration :300 μg / mL.
